# Synergistic apoptosis by combination of metformin and an *O*-GlcNAcylation inhibitor in colon cancer cells

**DOI:** 10.1186/s12935-023-02954-2

**Published:** 2023-06-02

**Authors:** Da Eun Lee, Geun Yong Lee, Hae Min Lee, Soo Young Choi, Su Jin Lee, Oh-Shin Kwon

**Affiliations:** 1 School of Life Sciences, BK21 FOUR KNU Creative BioResearch Group, Kyungpook National University, Daegu, 41566 Republic of Korea; 2 Department of Biomedical Science and Research Institute of Bioscience and Biotechnology, Hallym University, Chuncheon, 24252 Republic of Korea

**Keywords:** Apoptosis, Autophagy, Colon cancer, ER stress, Metformin, OSMI-1

## Abstract

**Background:**

Although autophagy is an important mediator of metformin antitumor activity, the role of metformin in the crosstalk between autophagy and apoptosis remains unclear. The aim was to confirm the anticancer effect by inducing apoptosis by co-treatment with metformin and OSMI-1, an inhibitor of *O*-GlcNAcylation, in colon cancer cells.

**Methods:**

Cell viability was measured by MTT in colon cancer cell lines HCT116 and SW620 cells. Co-treatment with metformin and OSMI-1 induced autophagy and apoptosis, which was analyzed using western blot, reverse transcription-polymerase chain reaction (RT-PCR) analysis, and fluorescence-activated cell sorting (FACS). Combined treatment with metformin and OSMI-1 synergistically inhibit the growth of HCT116 was confirmed by xenograft tumors.

**Results:**

We showed that metformin inhibited mammalian target of rapamycin (mTOR) activity by inducing high levels of C/EBP homologous protein (CHOP) expression through endoplasmic reticulum (ER) stress and activating adenosine monophosphate-activated protein kinase (AMPK) to induce autophagy in HCT116 cells. Interestingly, metformin increased *O*-GlcNAcylation and glutamine:fructose-6-phosphate amidotransferase (GFAT) levels in HCT116 cells. Thus, metformin also blocks autophagy by enhancing *O*-GlcNAcylation, whereas OSMI-1 increases autophagy via ER stress. In contrast, combined metformin and OSMI-1 treatment resulted in continuous induction of autophagy and disruption of *O*-GlcNAcylation homeostasis, resulting in excessive autophagic flux, which synergistically induced apoptosis. Downregulation of Bcl2 promoted apoptosis via the activation of c-Jun N-terminal kinase (JNK) and CHOP overexpression, synergistically inducing apoptosis. The activation of IRE1α/JNK signaling by OSMI-1 and PERK/CHOP signaling by metformin combined to inhibit Bcl2 activity, ultimately leading to the upregulation of cytochrome c release and activation of caspase-3.

**Conclusions:**

In conclusion, combinatorial treatment of HCT116 cells with metformin and OSMI-1 resulted in more synergistic apoptosis being induced by enhancement of signal activation through ER stress-induced signaling rather than the cell protective autophagy function. These results in HCT116 cells were also confirmed in xenograft models, suggesting that this combination strategy could be utilized for colon cancer treatment.

**Supplementary Information:**

The online version contains supplementary material available at 10.1186/s12935-023-02954-2.

## Introduction

Metformin is the most widely used drug to treat type 2 diabetes, but it also has antitumor activity against a variety of cancers, including colon cancer [1–3]. Although the tumor suppressor effect of metformin is well-established, the molecular mechanisms underlying this effect have not been elucidated. Recently, metformin has been shown to cause cell death in cancer cells by inducing endoplasmic reticulum (ER) stress [4, 5]. ER stress is caused by the accumulation of misfolded/unfolded proteins in the ER, which stimulate the activation of the unfolded protein response (UPR) to restore ER homeostasis. However, prolonged ER stress also activates apoptosis. Sensors for ER stress include inositol-requiring enzyme 1 α (IRE1α), protein kinase RNA-like endoplasmic reticulum kinase (PERK), and transcription factor 6 (ATF6), which control cell survival or death signals [6]. C/EBP homologous protein (CHOP) signaling via PERK can induce apoptosis by reducing the expression of Bcl2, an anti-apoptotic factor that releases cytochrome c and prevents the translocation of Bax to the mitochondria [7, 8]. IRE1α also plays a role in anticancer activity through the c-Jun N-terminal kinase (JNK) pathway [9]. The IRE1α/JNK pathway can also induce the release of cytochrome c through the phosphorylation of Bcl2, thus leading to apoptosis.

The mechanisms of apoptosis induced by ER stress are also related to autophagy. The crosstalk between autophagy and ER stress has been studied in-depth and these two systems are dynamically interconnected to either stimulate or inhibit each other [10]. Metformin induces autophagy by activating ER stress. PERK signaling can promote autophagy through the inhibition of mammalian target of rapamycin (mTOR) and contributes to autophagy through the IRE1α/XBP1 pathway [11, 12]. Phosphorylation of ULK1 by adenosine mono-phosphate-activated protein kinase (AMPK) is required for autophagy initiation and AMPK can promote autophagy by inhibiting the negative regulator mTORC1 [13]. The Beclin-1 complex is essential for phagocyte nucleation and vesicle elongation and includes the microtubule-associated proteins 1A/1B light chain 3 B (LC3) and SQSTM1/p62. After the cleavage of LC3 by LC3-I, phosphatidylethanolamine (PE) is conjugated in a multi-step process to form LC3-II, which is essential for autophagosome formation [14]. p62 acts as a docking receptor for the transport of degradation products. Mature autophagosomes, containing organelles and proteins, fuse with lysosomes, and their contents are broken down and recycled.

UDP-GlcNAc is an essential precursor of mammalian glycosylation that is produced by the hexosamine biosynthetic pathway and is primarily regulated by glucose-6-phosphate-glutamine:fructose-6-phosphate amidotransferase (GFAT) [15]. *O*-GlcNAcylation is a diverse and dynamic process in which *O*-linked *N*-acetylglucosamine is either transferred to the serine/threonine residues of intracellular proteins by *O*-GlcNAc transferase (OGT) or removed by *O*-GlcNAcase (OGA). Although *O*-GlcNAc is essential for cell survival, excessive increases in *O*-GlcNAc levels are also implicated in the pathogenesis of many chronic diseases, such as diabetic complications and the formation of tumor tissue, including breast, lung, and colon cancer [16]. Recent studies have shown that an increase in *O*-GlcNAcylation inhibits autophagy, moreover a decrease stimulates autophagic flux [17, 18]. As intracellular *O*-GlcNAcylation levels are significantly increased in most malignancies, they may be closely related to cancer progression. However, the regulatory mechanisms underlying increased *O*-GlcNAcylation and autophagy and their contribution to cancer proliferation, remain largely unknown.

Our previous study revealed that inhibition of *O*-GlcNAcylation sensitized HepG2 and HCT116 cells and was involved in apoptosis induction [19]. OSMI-1 potentially sensitizes trail-induced cell death in HCT116 cells through the blockade of NF-κB signaling and activation of apoptosis through ER stress response. In the present study, we investigated the anticancer effects that metformin exerts via the regulation of *O*-GlcNAcylation in HCT116 cells. This means that the mechanism of metformin in autophagic flux through AMPK remains unclear and the role of *O*-GlcNAcylation in ER stress response and autophagy needs to be elucidated. The present study showed that co-treatment with metformin and OSMI-1 synergistically induced apoptosis with an increase in autophagic flux in colon cancer cells.

## Materials and methods

### Cell culture and treatment

The HCT116 and SW620 human colon cancer cell lines were purchased from the Korean Cell Line Bank (Seoul, Korea). HCT116 was maintained in Dulbecco’s Modified Eagle Medium (DMEM) (Thermo Fisher Scientific, Waltham, MA, USA) supplemented with 25 mM NaHCO_3_, 10% fetal bovine serum (FBS) and 1% penicillin. SW620 was maintained in RPMI-1640 medium (Thermo Fisher Scientific) containing l-glutamine (300 mg/l) and supplemented with 10% FBS, and 1% penicillin. Cells were incubated at 37 °C in a humidified 5% CO_2_ atmosphere. Cells were cultured to 80% confluence and then treated with metformin (Sigma-Aldrich, St. Louis, MO, USA) and OSMI-1 (Cayman Chemical, Ann Arbor, MI, USA). For some experiments, the cells were treated with inhibitors for 1 h before treatment with metformin and/or OSMI-1. The mCherry-EGFP-LC3B plasmid was obtained from Addgene (Watertown, MA, USA). The CHOP siRNA (Invitrogen, Waltham, MA, USA) sequence was designed as follows: 5′-GCCUGGUAUGAGGACCUGC-3′. Cells were transiently transfected with the plasmid or target siRNA using Lipofectamine 2000 (Invitrogen), according to the manufacturer’s instructions.

### Preparation of protein extraction and immunoblot analysis

HCT116 and SW620 cells were lysed by cell lysis solution (1× PBS, 1% Nonidet P40, and 1 mM EDTA) containing a protease inhibitor cocktail (Roche, Mannheim, Germany). Equal amounts of protein were diluted in 2× sodium dodecyl sulfate (SDS) loading dye, separated on 8–15% SDS-polyacrylamide (SDS-PAGE) gels, and transferred onto nitro-cellulose membranes (GE Healthcare Life Sciences, Boston, MA, USA). After blocking with 5% skim milk at room temperature for 1 h, the membranes were probed overnight with primary antibodies at 4 °C. Anti-*O*-GlcNAc antibody was purchased from Sigma-Aldrich. An anti-PARP antibody was purchased from Invitrogen. Anti-cleaved caspase-3, IRE1α, P-eIF2α, eIF2α, CHOP, P-AMPK, AMPK, P-ULK, P-mTOR, mTOR, P-p70 S6K, p70 S6K, ATG5, and LC3B antibodies were purchased from Cell Signaling Technology (Danvers, MA, USA). Anti-ULK, PERK, ATF6, ATF4, P-JNK, JNK, Bax, P-Bcl2, Bcl2, p62, GFAT, caspase-8, and β-actin were purchased from Santa Cruz Biotechnology (Santa Cruz, CA, USA). Membranes were washed with TBST and incubated with horseradish peroxidase (HRP)-conjugated anti-mouse or anti-rabbit immunoglobulin G secondary antibodies at room temperature for 30 min. Immunoreactive bands were developed using an ECL Plus Detection System (GE Healthcare Life Sciences) and the bands were quantified using ImageJ software (NIH, USA).

### Immunofluorescence (IF)

Cells on coverslips were washed in TBST for 10 min and fixed in Fix & Perm Reagent A (Fixation Medium, Invitrogen) for 15 min. The cells were then washed in TBST for 10 min and fixed with Perm Reagent B (permeabilization medium) for 15 min. After blocking with 1% BSA containing 0.01% goat serum at room temperature for 1 h, the cells were incubated with primary antibodies (LC3B, 1:200, Cell Signaling Technology) overnight at 4 °C. The cells were washed with TBST and incubated with Alexa-conjugated secondary antibodies (1:200, Invitrogen) at room temperature for 1 h in the dark. The coverslips were washed with TBST and mounted using ProLong Gold mounting medium (Invitrogen). Images were taken by a confocal fluorescence microscope (FV1200; Olympus, Tokyo, Japan).

### Cell viability assay

HCT116 (2 × 10^3^ cells/well) and SW620 (5 × 10^3^ cells/well) cells were seeded in 96-well microplates and incubated for 24 h [20]. And then cells were treated with various concentrations of metformin (1, 5, 10, 20, and 25 mM) for 48 h. Cell viability was measured using the 3-(4,5-dimethylthiazol-2-yl)-2,5-diphenyl-tetrazolium bromide (MTT) assay. After metformin treatment, 10 µl of MTT solution (1 mg/ml) was added to each well, and the cells were incubated for an additional 4 h. MTT solution containing phenol red was removed. Dimethyl sulfoxide (DMSO) was added to each well (100 µl) and the absorbance value was measured at 580 nm with a spectrophotometric microplate reader (Molecular Devices, San Jose, CA, USA). The results were expressed as the percentage of cell viability.

### RNA isolation and reverse transcription-polymerase chain reaction (RT-PCR)

Total cellular RNA was isolated from HCT116 cells using TRIzol reagent (Invitrogen) according to the manufacturer’s instructions. The extracted RNA was quantified using a NanoDrop spectrophotometer (DeNoVIX Inc., Wilmington, DE, USA). cDNA synthesis was performed using a LAVO pass cDNA synthesis kit (Cosmo Genetech, Seoul, Korea) according to the manufacturer’s instructions. XBP1 was amplified Using TaKaRa Ex Taq (TaKaRa, Otsu, Japan) with primers XBP1 forward: 5′-GGTCTGCTGAGTCCGCAGCAGG-3′, reverse: 5′-GAAAGGGAGGCTGGTAAGGAAC-3′. PCR amplicons were electrophoresed on a 1.5% agarose gel.

### Cytochrome C release assay

Cells (1 × 10^5^) were harvested and suspended in 100 μl digitonin (50 μg/ml in 1 × PBS with 100 mM KCl) on ice for 3–5 min until more than 95% of the cell were permeabilized according to a trypan blue positive stain. Permeabilized cells were fixed directly in 100 μl 4% paraformaldehyde for 20 min at room temperature and immediately centrifuged (5 min at 500 g). After three washes in 1× PBS, cells were incubated in a blocking buffer (3% BSA, 0.05% saponin in 1× PBS) for 1 h and centrifuged at 3000×*g* for 5 min. The pellet was incubated overnight at 4 °C with 1:200 cytochrome c antibody (Santa Cruz) in a blocking buffer. After three washes in 1× PBS, cytochrome c was visualized with 1:200 Alexa 488 secondary antibody (Invitrogen) in a blocking buffer at room temperature for 1 h. The cells were then analyzed using flow cytometry (BD Biosciences, Franklin Lakes, NJ, USA) to detect FITC-A.

### Annexin V-fluorescein isothiocyanate (FITC)/propidium iodide (PI) flow cytometric assay

Cell apoptosis induced by metformin and OSMI-1 was detected and quantified using annexin V-FITC/PI apoptosis detection kits (Invitrogen). Briefly, HCT116 cells were seeded at a density of 2 × 10^5^ cells/ml in cell culture dishes. After treating cells with 25 mM metformin with or without 20 µM OSMI-1 for 48 h, they were trypsinized, washed once with 1× PBS, and immediately suspended in 1× annexin V binding buffer (100 µl). Then the cells were incubated with annexin V-FITC (5 μl) and allowed to react at room temperature for 15 min. After incubation, PI (0.1 μl) was added to each tube and the mixture was then placed on ice at 4 °C in the dark. Finally, 1× annexin V binding buffer (400 μl) was added to each tube and the samples were analyzed by fluorescence-activated cell sorting (FACS) (BD Biosciences).

### Tumor xenograft and growth

All animal study was approved by the Animal Care and Use Committee of the Kyungpook National University, Korea (2021-0208). Female BALB/c-Foxn1nu/ArcGem nude mice aged 5 weeks (GEM Biosciences, Cheongju, Republic of Korea) were injected subcutaneously with 5 × 10^6^ HCT116 cells into the flank of each mouse. When the tumors volumes approximately reached 90–110 mm^3^, tumor-bearing mice were randomly divided into four treatment groups (n = 7/group) as follows: control (DMSO), metformin (200 mg/kg/day, intraperitoneally), OSMI-1 (1 mg/kg/daily, intravenously) and combination of metformin and OSMI-1. Tumor length and width were measured with a digital caliper according to a score sheet that recorded tumor size, which was calculated using the following formula: length × width^2^ × 0.5 (mm^3^). When after injection of 3 weeks, mice were sacrificed and tumors were subjected to western blot analysis.

### Statistical analysis

All experiments are presented as the mean ± SEM. Two-sample t-tests were per-formed to evaluate the differences between groups. All experiments were repeated at least 3–5 times. Statistical analysis was performed using the Student’s t-test for comparison between groups and P-values < 0.05 were considered statistically significant. All data analysis was performed using SPSS software (IBM, USA).

## Results

### Metformin induces autophagy via the AMPK pathway in HCT116 cells

**Fig. 1:**
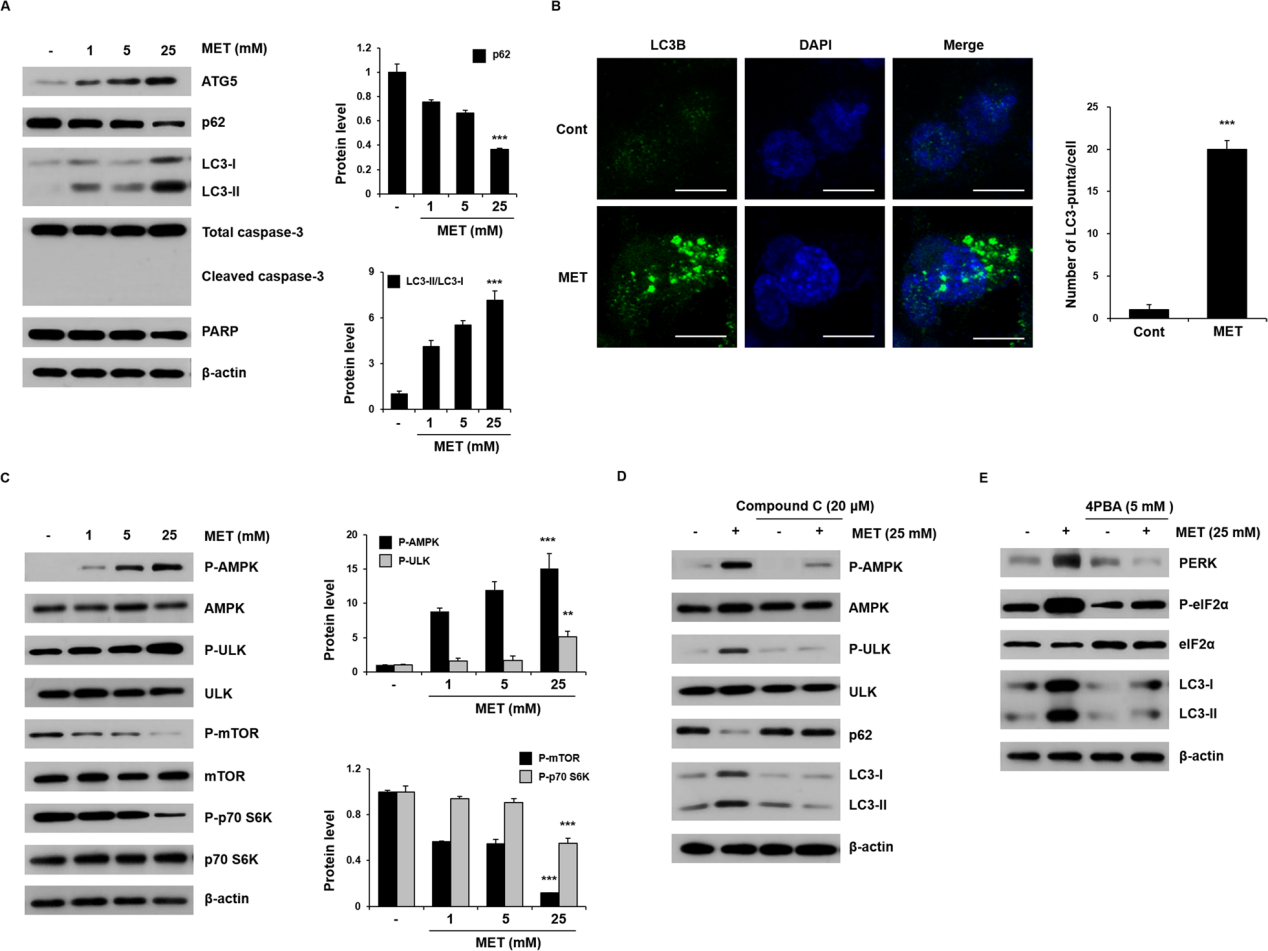
Metformin-mediated autophagy via the AMPK pathway. **A** HCT116 cells were treated with metformin (1, 5, and 25 mM) for 48 h. The levels of ATG5, p62, LC3-I, LC3-II, cleaved caspase-3, and PARP were examined by western blot analysis. β-Actin was used as a loading control in all western blot analyses. **B** HCT116 cells were treated with metformin (25 mM) for 48 h, followed by immunofluorescence analysis. The localization of LC3B (green) was visualized using a confocal microscope (original magnification ×1000, scale bar = 10 μm). (C) HCT116 cells were treated with metformin (1, 5, and 25 mM) for 48 h and then the levels of P-AMPK, P-ULK, P-mTOR, and P-p70 S6K were investigated by western blot analysis. **D** HCT116 cells were pretreated for 1 h in the presence or absence of Compound C (20 μM), followed by further treatment in the presence or absence of metformin (25 mM) for 48 h. The levels of P-AMPK, P-ULK, p62, LC3-I, and LC3-II were investigated by western blot analysis. **E** HCT116 cells were cultured for 1 h in the presence or absence of 4PBA (5 mM) and then treated for 48 h in the presence or absence of metformin (25 mM). The levels of PERK, P-eIF2α, LC3-I, and LC3-II were investigated by western blot analysis. **A**–**C** Significance was determined by control and 25 mM metformin group Student’s t-test. **p < 0.01, ***p < 0.001

Although autophagy is known to participate in drug resistance in cancer, it is not known whether metformin mediates drug resistance through autophagy. In the present study, when HCT116 cells were treated with metformin (1, 5, and 25 mM) for 48 h (Fig. 1A), the level of p62 protein decreased slightly in a concentration-dependent manner, but the expression of ATG5 and LC3-II significantly increased compared to in untreated cells. To determine the potential effect of metformin on apoptosis, we evaluated the rate of apoptosis by measuring the activation of cleaved caspase-3 and PARP with metformin treatment. Apoptosis marker proteins expression was mostly unchanged in a concentration-dependent metformin treatment. Treatment with 25 mM metformin for 48 h significantly increased the number of LC3-puncta in HCT116 cells (Fig. 1B). These results indicate that metformin induces autophagy, leading to the formation of autophagosomes.

To determine whether metformin-induced autophagy occurs through the AMPK signaling pathway, HCT116 cells were treated with metformin (1, 5, and 25 mM) for 48 h and analyzed using western blotting (Fig. 1C). Compared with the control group, the expression levels of P-AMPK (Thr172) and P-ULK (Ser555) were significantly increased with metformin treatment, indicating that AMPK was activated. mTOR regulates cell growth and negatively regulates autophagy. Levels of P-mTOR (Ser2448) and P-p70 S6 kinase (Thr421/Ser424) were significantly reduced in a metformin concentration-dependent manner.

To determine whether metformin-mediated AMPK activation is directly linked to the autophagy process, HCT116 cells were treated with the AMPK inhibitor Compound C (Fig. 1D). Blocking the activation of AMPK significantly reduced the level of P-ULK and LC3-II that increased following metformin treatment, but the level of p62 did not decrease, indicating that autophagic flux was blocked. Additional experiments were conducted to confirm whether or not autophagy was induced through AMPK. To confirm autophagic flux, we transfected HCT116 cells with mCherry-GFP-LC3B (Additional file 1: Fig. S1). When the autophagy process proceeds, compared to the control, the red and green signals merge to show yellow fluorescence. When treated with metformin, it was observed that yellow dots appeared, and when Compound C and metformin were co-treated, it was confirmed that yellow fluorescence did not appear.

Next, we investigated the relationship between autophagy and ER stress. Metformin increased the expression of PERK and downstream target phosphorylation of eIF, which precedes LC3B (Fig. 1E). Treatment with the ER stress inhibitor 4-phenyl butyric acid (4PBA) alone did not affect the PERK, P-eIF2α, or LC3B, but treatment with 4PBA in combination with metformin downregulated these levels. These results suggest that metformin induces ER stress, which can be inhibited by 4PBA. Taken together, these results indicate that metformin induces ER stress-dependent autophagy in HCT116 cells.

### In HCT116 cells, metformin increases *O*-GlcNAcylation, and inhibition of *O*-GlcNAcylation promotes autophagy

**Fig. 2:**
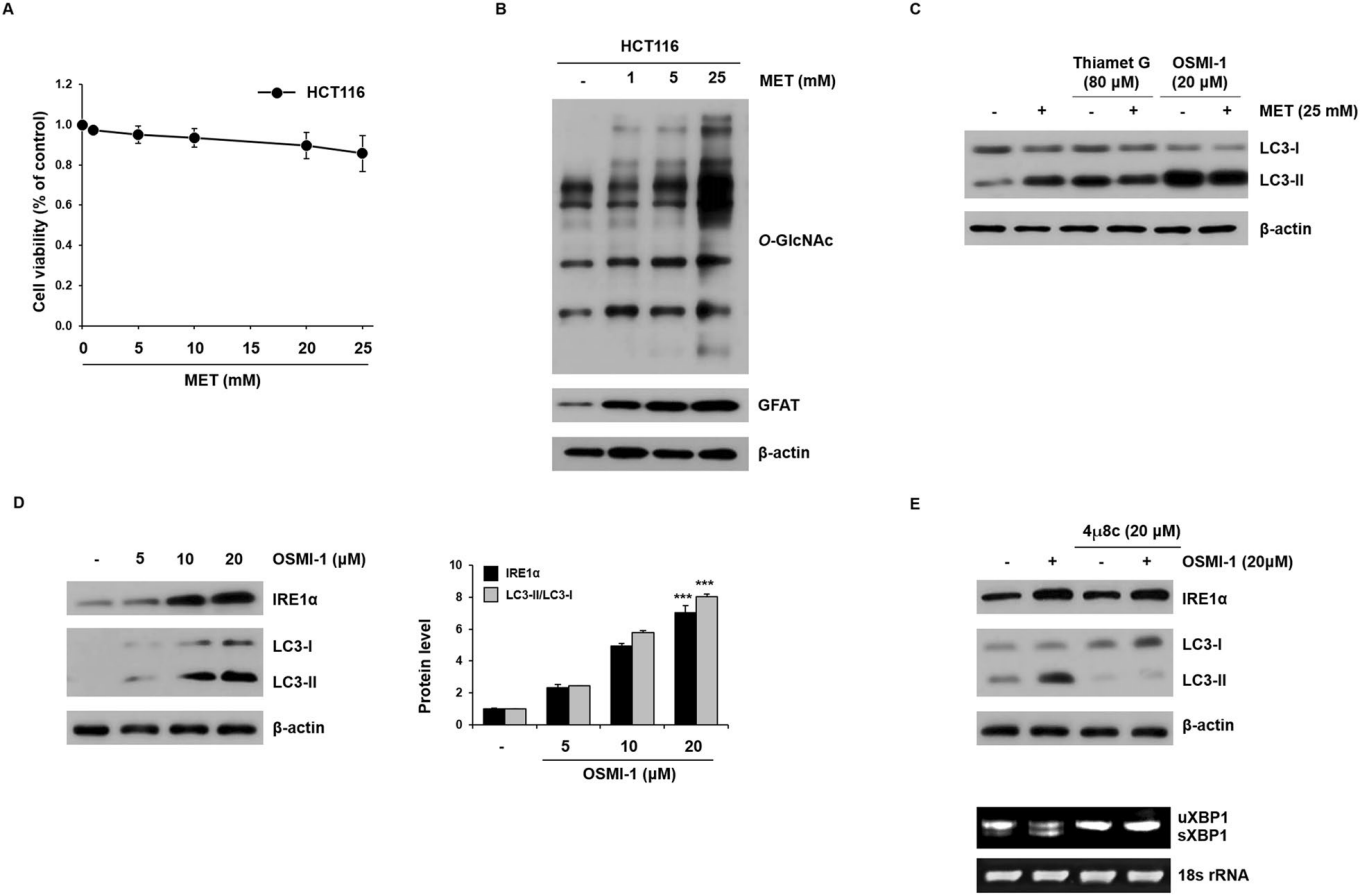
Enhancement of autophagic flux by blockade of *O*-GlcNAcylation. **A** HCT116 cells were incubated with metformin (1, 5, 10, 20, and 25 mM) for 48 h, and cell viability was determined by MTT assay. **B** HCT116 cells were treated with metformin (1, 5, and 25 mM) for 48 h, the levels of *O*-GlcNAc and GFAT were analyzed by western blot. β-Actin was used as a loading control in all western blot analyses. **C** HCT116 cells were cultured in the presence or absence of Thiamet G (80 μM) or OSMI-1 (20 μM) and then treated with or without metformin (25 mM) for 48 h. The levels of LC3-I and LC3-II were determined by western blot analysis. **D** HCT116 cells were treated with various concentrations of OSMI-1 (5, 10, and 20 μM) for 48 h, and the levels of IRE1α, LC3-I, and LC3-II were investigated by western blot analysis. **E** HCT116 cells were cultured for 1 h in the presence or absence of 4µ8c (20 µM) and then treated for 48 h with or without OSMI-1 (20 µM). The levels of IRE1α, LC3-I, and LC3-II were investigated by western blot analysis (top). Levels of sXBP1 were determined by RT-PCR using primers to amplify both unspliced and spliced mRNA species (bottom). 18 s rRNA was used as the loading control for RT-PCR. **A**, **D** Significance was determined by control and 20 μM OSMI-1 group Student’s t-test. ***p < 0.001

We evaluated the concentration-dependent cytotoxic effects of metformin on HCT116 or SW620 cells by performing an MTT assay. As shown in Fig. 2A, there was no significant decrease in cell viability even with 25 mM metformin treatment in HCT116 cells. In contrast, metformin showed a marked cytotoxic effect on SW620 cells, significantly reducing the number of viable cells (Additional file 1: Fig. S2A).

Recent studies have revealed that *O*-GlcNAcylation is involved in the regulation of autophagy. We first investigated the association between metformin-dependent *O*-GlcNAcylation in colon cancer cells. Metformin treatment for 48 h significantly increased the level of *O*-GlcNAcylated proteins in HCT116 cells in a dose-dependent manner and also upregulated the levels of GFAT (Fig. 2B). However, a dose-dependent effect of metformin on *O*-GlcNAcylation was not observed in SW620 cells and the level of GFAT did not change (Additional file 1: Fig. S3).

To determine how the metformin-induced increase in *O*-GlcNAcylation occurs and whether it is related to autophagy, HCT116 cells were treated with metformin and Thiamet G, an OGA inhibitor, or OSMI-1, an OGT inhibitor (Fig. 2C). The level of LC3-II was slightly increased by metformin; however, there was no discernable effect of additional treatment with Thiamet G. In contrast, OSMI-1 significantly increased the level of LC3-II and the ratio of LC3-II/LC3-I was further increased. Taken together, the metformin-induced increase in *O*-GlcNAcylation in HCT116 cells negatively affected the autophagy process. However, reducing of *O*-GlcNAcylation by OSMI-1 promoted autophagy in HCT116 cells. HCT116 cells were treated with OSMI-1 (5, 10, and 20 μM) for 48 h, the expression levels of *O*-GlcNAc decreased in a concentration-dependent manner compared to the control group (Additional file 1: Fig. S4).

The ER stress signaling pathway is involved in cellular autophagy and IRE1α can promote the initiation of autophagy. As shown in Fig. 2D, when HCT116 cells were treated with OSMI-1 (5, 10, and 20 μM) for 48 h, the expression levels of IRE1α and LC3-II increased in a concentration-dependent manner compared to the control group. However, metformin did not show the expression levels of IRE1α increased in a dose-dependent effect (Additional file 1: Fig. S5).

In contrast, treatment with the XBP1 inhibitor 4μ8C did not affect IRE1α protein expression but downregulated the expression level of LC3-II protein (Fig. 2E, top). Similarly, the splicing level of XBP1 (sXBP1) increased following OSMI-1 treatment, whereas treatment with 4μ8C inhibited the increase in sXBP1 caused by OSMI-1 treatment (Fig. 2E, bottom). These results suggest that OSMI-1 induces IRE1α/XBP1 signaling and is associated with autophagy. These results indicate that a decrease in cellular *O*-GlcNAcylation may increase autophagy through ER stress and the IRE1α/XBP1 pathway.

### Co-treatment with metformin and OSMI-1 induces synergistic apoptosis by combining CHOP and JNK signaling, respectively

**Fig. 3:**
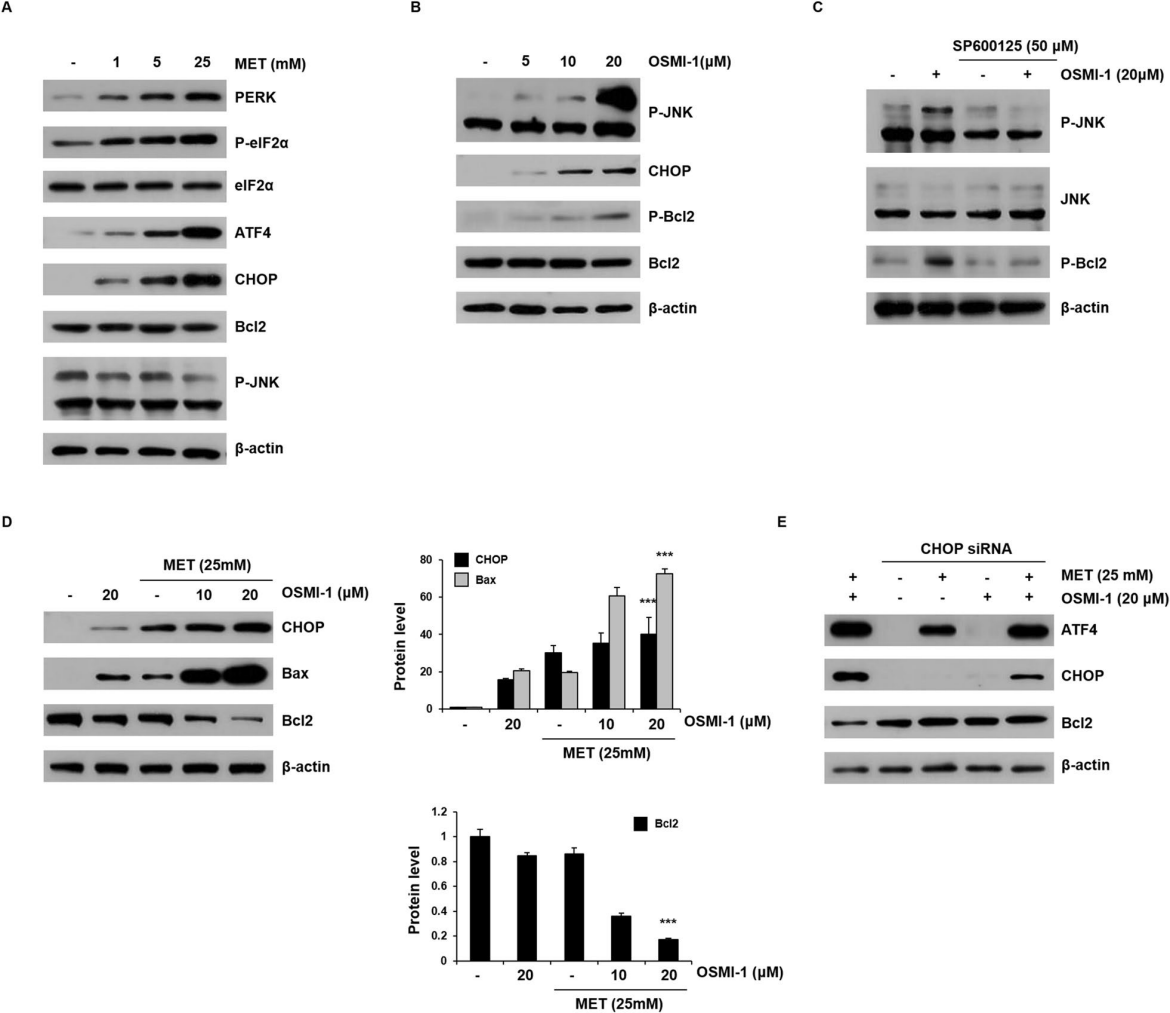
Metformin and OSMI-1 induce inhibition of Bcl2 activity through CHOP and JNK signaling pathways, respectively. **A** HCT116 cells were treated with metformin (1, 5, and 25 mM) for 48 h and the levels of PERK, P-eIF2α, ATF4, CHOP, Bcl2, and P-JNK were analyzed by western blot analysis. β-Actin was used as a loading control in all western blot analyses. **B** HCT116 cells were treated with OSMI-1 (5, 10, and 20 μM) for 48 h and the levels of P-JNK, CHOP, P-Bcl2, and Bcl2 were investigated by western blot analysis. **C** HCT116 cells were pretreated for 1 h in the presence or absence of SP600125 (50 µM) and then treated for 48 h with or without OSMI-1 (20 µM). The levels of P-JNK and P-Bcl2 were investigated by western blot analysis. **D** HCT116 cells were treated with metformin (25 mM), OSMI-1 (10 μM or 20 μM), or a combination thereof for 48 h, and CHOP, Bax, and Bcl2 levels were determined by western blot analysis. Significance was determined by control and combination treatment group Student’s t-test. ***p < 0.001. **E** HCT116 cells in 6-well plates were transfected with CHOP siRNA and treated with metformin (25 mM), OSMI-1 (20 μM), or a combination thereof for 48 h. The levels of ATF4, CHOP, and Bcl2 were determined by western blot analysis

To determine whether metformin induces ER stress, we next investigated the expression of ER stress marker proteins, including PERK. As shown in Fig. 3A, PERK, P-eIF2α, ATF4, and CHOP levels showed a dose-dependent increase in response to metformin treatment. CHOP induces apoptosis by decreasing the levels of Bcl2. However, changes in Bcl2 and P-JNK levels with increasing metformin concentration were not observed. Furthermore, the IRE1α pathway did not appear to be activated in HCT116 cells treated with metformin alone, since the splicing distribution of XBP1 was not affected (Additional file 1: Fig. S6). In contrast, the induction of ER stress by OSMI-1 resulted in the activation of P-JNK via IRE1α. P-JNK activity increased significantly in a dose-dependent manner with OSMI-1 treatment and CHOP and P-Bcl2 levels were also slightly increased (Fig. 3B). Similar to metformin treatment, OSMI-1 treatment did not cause a significant decrease in Bcl2 expression. To determine whether OSMI-1-mediated JNK activation is directly linked to Bcl2, HCT116 cells were treated with the JNK inhibitor SP600125 (Fig. 3C). Blocking the activation of JNK significantly reduced the level of P-Bcl2 that was induced by OSMI-1. This suggests that the IRE1α/JNK pathway blocked the activity of Bcl2 to induce apoptosis.

We confirmed whether ER stress-mediated pathways are active in the treatment of metformin and/or OSMI-1 (Additional file 1: Fig. S7). ER stress marker proteins IRE1α, PERK, and ATF6 expression levels were increased in co-treatment with metformin and OSMI-1. Interestingly, co-treatment of HCT116 cells with metformin and OSMI-1 significantly increased the expression of CHOP and Bax and decreased the level of Bcl2 (Fig. 3D). The role of CHOP in the reduction of Bcl2 levels, which leads to apoptosis, was confirmed using siRNA. As shown in Fig. 3E, in HCT116 cells treated with metformin and OSMI-1, siRNA resulted in a significant decrease in CHOP expression and restored the level of Bcl2. This suggests that CHOP induction through PERK/ATF4 signaling plays a role in triggering apoptosis.

**Fig. 4:**
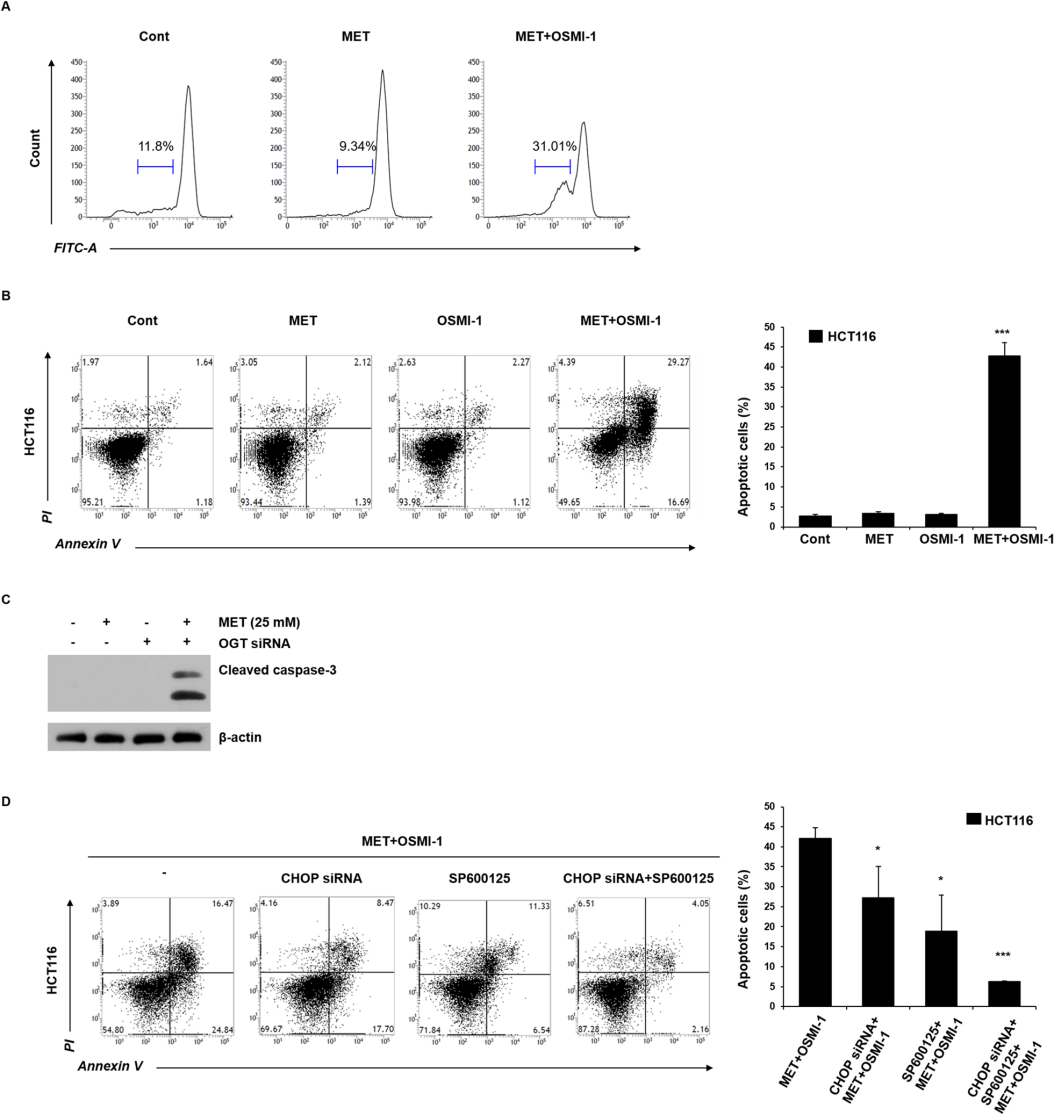
Induction of apoptosis through cytochrome c release with combined metformin and OSMI-1 treatment. **A** Flow cytometry analysis of cytochrome c release showing the fluorescence intensity of the FITC-A channel in HCT116 cells. Cells were treated with metformin (25 mM) alone or in combination with OSMI-1 (20 μM) for 48 h and the amount of cytochrome c was analyzed by flow cytometry. **B** HCT116 cells were treated with metformin (25 mM), OSMI-1 (20 μM), or a combination thereof for 48 h. After staining with Annexin V-FITC/PI, the level of apoptosis was determined by flow cytometry. The proportion of early and late apoptotic cells was compared. **C** HCT116 cells were transfected with OGT siRNA and treated with metformin (25 mM) alone or a combination thereof for 48 h. The level of cleaved caspase-3 was determined by western blot analysis. β-Actin was used as a loading control in all western blot analyses. **D** HCT116 cells were transfected with CHOP siRNA and incubated with SP600125 alone or a combination thereof for 1 h and then treated with a combination of metformin (25 mM) and OSMI-1 (20 μM) for 48 h. After staining with annexin V-FITC/PI, the level of apoptosis was determined by flow cytometry. The proportion of early and late apoptotic cells was determined. **B**, **D** Experiments were performed in triplicate and data represents the mean ± SEM. Significance was determined by control and combination group Student’s t-test. ***p < 0.001

We measured the apoptotic toxic effects of metformin and OSMI-1 in HCT116 cells by determining the extent of cytochrome c release. As shown in Fig. 4A, the release of cytochrome c was dramatically increased in the combination group compared with the control group. To determine whether metformin and OSMI-1 treatment led to apoptosis, the apoptosis rate was measured by flow cytometry after staining with annexin V and PI. As shown in Fig. 4B, the effects of metformin and OSMI-1 alone were limited. However, the combination treatment significantly increased the rate of cell death, resulting in > 40% cell death compared to the individual treatments.

To confirm whether OGT knockdown enhances the combination treatment effect, metformin and OGT siRNA were treated instead of OSMI-1. Combined treatment of metformin and OGT siRNA increased cleaved caspase-3 with the same effect as the combination of metformin and OSMI-1 (Fig. 4C). In addition, the synergistic effect through the combination of metformin and OSMI was also confirmed in liver cancer cells (Additional file 1: Fig. S8). We next determined whether apoptosis caused by the co-administration of metformin and OSMI-1 occurs via a CHOP- and JNK-dependent pathway. HCT116 cells were transfected with CHOP siRNA for 24 h or pretreated with SP600125 for 1 h and then co-treated with metformin and OSMI-1 for 48 h, confirmed by flow cytometry (Fig. 4D). The apoptosis rates of cells transfected with CHOP siRNA or pretreated with SP600125 were 27% and 19%, respectively. The apoptosis rates of cells transfected with CHOP siRNA and pretreated with SP600125 group was significantly reduced to 6%. Taken together, it is suggested that activation of the IRE1α/JNK pathway is enhanced by inhibition of *O*-GlcNAcylation and that metformin-induced PERK/CHOP signaling is combined to promote apoptosis.

### Autophagic flux and apoptosis are mutually regulated in HCT116 cells

**Fig. 5:**
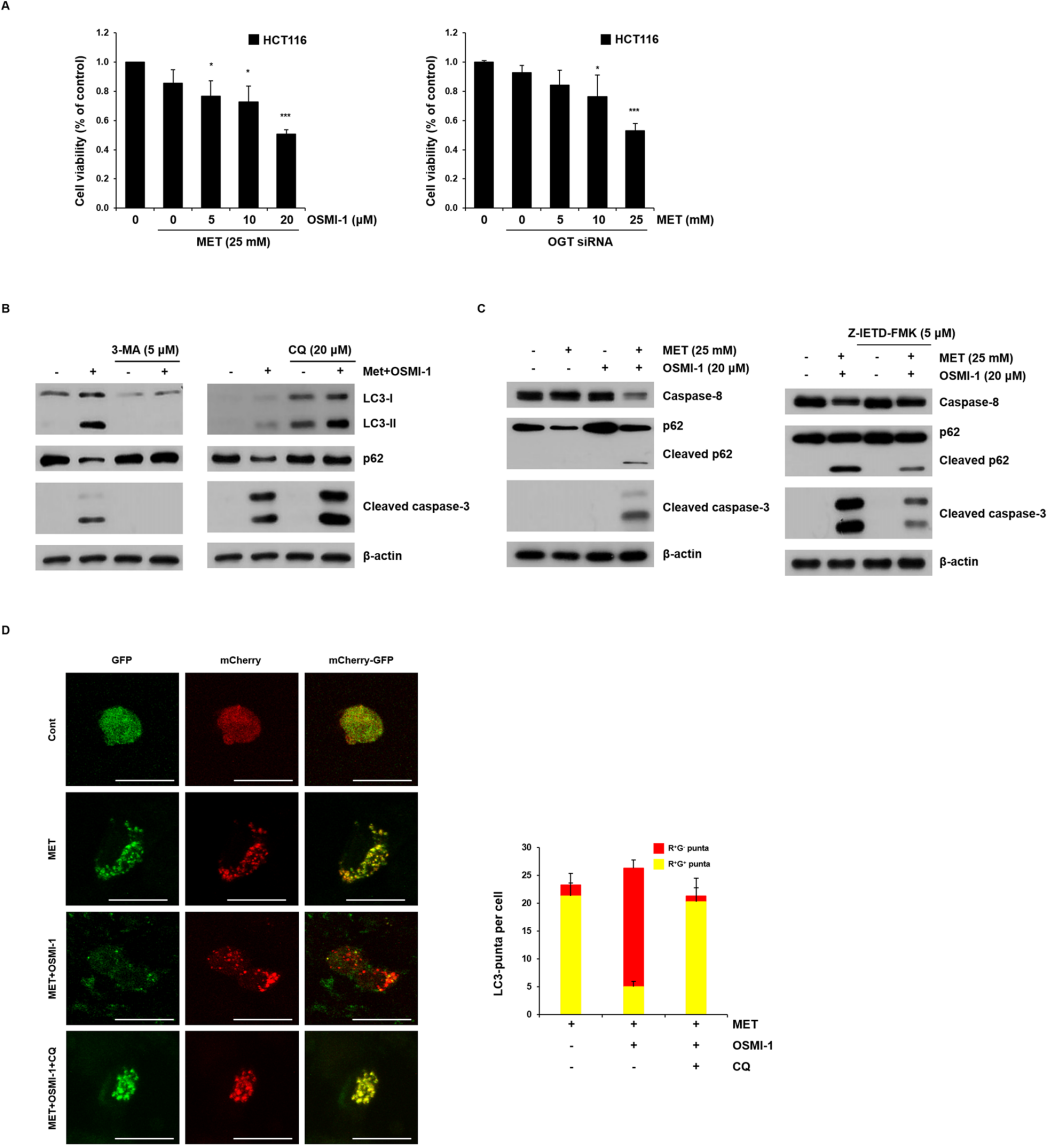
Regulation of apoptosis through autophagy. **A** HCT116 cells were treated with OSMI-1 (5, 10, and 20 μM) and metformin (25 mM) for 48 h, and the cell viability was determined by MTT assay (left). The cell viability of cells transfected with OGT siRNA and treated with metformin (5, 10, and 25 mM) for 48 h was determined by MTT assay (right). Significance was determined by control and combination group Student’s t-test. *p < 0.05, ***p < 0.001. **B** HCT116 cells were pretreated with 3-MA (5 μM) (left) or CQ (20 μM) (right) for 1 h and then treated with metformin and OSMI-1 for 48 h. The levels of LC3-I, LC3-II, p62, and cleaved caspase-3 were determined by western blot analysis. β-Actin was used as a loading control in all western blot analyses. **C** HCT116 cells were treated with metformin (25 mM), OSMI-1 (20 μM), or a combination for 48 h, and then the levels of caspase-8, p62, and cleaved caspase-3 were determined by western blot analysis (left). To confirm the role of caspase-8, HCT116 cells were pretreated with or without Z-IETD-FMK for 1 h and then treated with a combination of metformin (25 mM) and OSMI-1 (20 μM) for 48 h. The levels of caspase-8, p62, and cleaved caspase-3 were analyzed by western blot analysis (right). **D** HCT116 cells were transfected with the mCherry-EGFP-LC3 plasmid for 24 h and treated with metformin (25 mM) alone or in combination with OSMI-1 (20 μM) for 48 h. The CQ-treated group was transfected with the plasmid and then pretreated with CQ (20 μM) for 1 h, followed by treatment with metformin (25 mM) and OSMI-1 (20 μM) for 48 h. Immunofluorescence staining of LC3 was visualized using a confocal laser scanning microscope (original magnification ×1000, scale bar = 10 μm). Data represent the mean ± SEM

We performed an MTT assay to evaluate the viability of HCT116 cells in the combined treatment with metformin and OSMI-1 (Fig. 5A). Compared to the control group, there was no significant change in the survival rate even after treatment with 25 mM metformin alone. However, when metformin and OSMI-1 were used in combination, cell viability significantly decreased in a concentration-dependent manner (Fig. 5A, left and Additional file 1: Fig. S9). The same effect was observed when OGT siRNA was used instead of OSMI-1 (Fig. 5A, right). This suggests that intracellular *O*-GlcNAcylation levels are related to cytoprotective functions.

We next investigated whether the apoptosis rate of cells induced by the combination of metformin and OSMI-1 was related to autophagic flux in HCT116 cells. HCT116 cells were pretreated with 3-MA, a pan-PI3K inhibitor, for 1 h, followed by simultaneous treatment with metformin and OSMI-1 (Fig. 5B, left). Unlike the control group, early autophagy was blocked in the treated group, so there was no increase in LC3-II and caspase-3 activation and no decrease in p62 did not occur. To further elucidate the role of autophagy in metformin and OSMI-1 co-treated HCT116 cells, the cells were treated with CQ, an autophagy inhibitor that protects lysosomal acidification or inhibits fusion between autophagosomes and lysosomes. Treatment with CQ alone increased the level or accelerated the accumulation of LC3-II compared to the controls. In contrast, co-treatment with CQ did not block the activation of caspase-3 but rather resulted in slightly more cleavage of caspase-3 and expression of LC3-II (Fig. 5B, right). Therefore, these results suggest that apoptosis affected by autophagic flux was regulated in the early stages of autophagy in HCT116.

Next, an experiment was performed to confirm the relationship between caspase-8 involved in apoptosis and autophagy (Fig. 5C, left). Unlike single treatment, the combined treatment of metformin and OSMI-1 cleaves and activates caspase-8, which in turn cleaves and inactivates p62, whereas caspase-3 is activated. To confirm the role of caspase-8, we combined metformin and OSMI-1 with or without Z-IETD-FMK, a caspase-8 inhibitor, and confirmed it by western blot analysis (Fig. 5C, right). In HCT116 cells, the level of cleaved p62 was proportional to whether caspase-8 was inhibited, suggesting that the level of cleaved p62 in autophagy is linked to the apoptosis mechanism.

To track autophagic flux, LC3B was fluorescently tagged with mCherry-GFP-LC3B and transfected into HCT116 cells (Fig. 5D). Fluorescent proteins exhibit yellow fluorescence due to overlapping red and green signals in the initial autophagy process, but are unstable in acidic environments such as autolysosomes and thus emit only red fluorescence. The number of yellow dots in the group treated with metformin alone was significant compared to the control group, but more red fluorescence was observed in the group treated with metformin and OSMI-1, indicating that autophagic flux was increased. In addition, in the combination group pretreated with CQ, we observed widely distributed yellow spots, indicating that the late autophagy process was blocked. Taken together, these results suggest that regulation of autophagic flux in HCT116 cells is correlated with apoptosis.

### Combination treatment with metformin and OSMI-1 synergistically inhibits the growth of HCT116 xenografted tumors

**Fig. 6:**
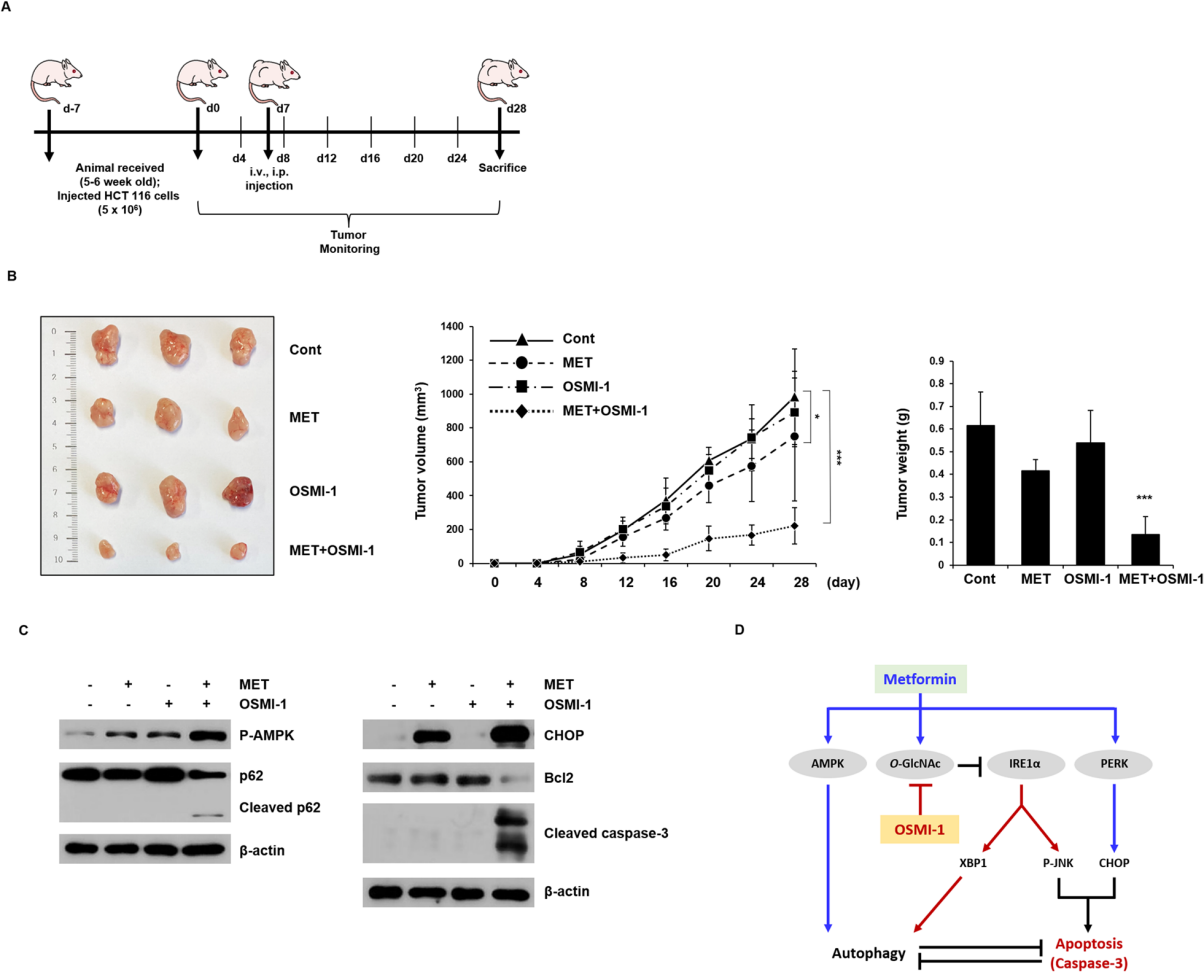
Inhibition of growth and apoptosis of xenograft tumors by combination treatment of metformin and OSMI-1. **A** BALB/c nude female mice were inoculated subcutaneously with HCT116 cell administration vehicle (DMSO), metformin, OSMI-1 or a combination and tumor growth was monitored for 28 days. **B** Representative images of subcutaneous xenograft tumors in nude mice treated with metformin, OSMI-1, or a combination thereof (left). weight measurements at endpoints are presented as the mean ± SEM (right). Volumes of xenograft tumors in metformin, OSMI-1, and combination treatment groups at the indicated time points were measured and growth profiles were expressed as mean ± SEM (middle). The results of tumor weight measurements at endpoints are presented as mean ± SEM (right). Significance was determined by control and combination group Student’s t-test. *p < 0.05, ***p < 0.001. **C** Expression levels of proteins involved in autophagy (P-AMPK and p62) were determined by western blot analysis (left). The levels of proteins involved in apoptosis (CHOP, Bcl2, and cleaved caspase-3) were also determined by western blot, and β-actin was used as the loading control (right). **D** Proposed signaling pathways for the activity of metformin and OSMI-1. The activation pathways of metformin and OSMI-1 in HCT116 cells are shown in blue and red, respectively. Combination therapy induces apoptosis through caspase activation rather than cytoprotective autophagy. Arrows and bars indicate activation and inhibition, respectively

Next, we investigated the antitumor effects of metformin and OSMI-1 on HCT116 cells using a xenograft mouse model. One week after the transplantation of HCT116 cells, tumor-bearing mice were randomly divided and metformin (200 mg/kg/day) and OSMI-1 (1 mg/kg/day) were administered alone or in combination by intraperitoneal or intravenous injection (Fig. 6A).

As a result, as shown in Fig. 6B, the extracted tumor volume and tumor weight slightly decreased in the single administration group compared to the control group (983.9 ± 281.8 mm^3^ and 0.62 ± 0.15 g, respectively), whereas the concurrent administration group showed a significant decrease (222.1 ± 105.9 mm^3^ and 0.14 ± 0.08 g) (Additional file 1: Fig. S10). Similarly, concurrent administration group also decreased tumors in the HCT116 p53^−/−^ xenograft mouse model (Additional file 1: Fig. S11). Therefore, the antitumor effects of metformin and OSMI-1 on HCT116 cells do not appear to be related to TP53. In addition, the expression levels of autophagy, ER stress, and apoptosis-related markers in tumor xenograft tissues were detected by western blot analysis (Fig. 6C). In the group treated with metformin and OSMI-1 in combination, the activation level of P-AMPK in the tumor tissue was significantly increased and the expression level of cleaved p62 was also significantly increased, confirming that fragments were generated. The expression level of CHOP showed a metformin-specific increase and it increased more synergistically in the combined administration group. On the other hand, simultaneous treatment with metformin and OSMI-1 significantly decreased Bcl2 levels and increased cleaved caspase-3. These data suggest that co-administration of OSMI-1 with metformin significantly reduced the growth of HCT116 xenografted tumors via autophagic flux and apoptosis, which is consistent with the previously mentioned in vitro results of this study.

Here, we propose the following apoptosis mechanism in HCT116 cells. Metformin activates the PERK/CHOP pathway that induces apoptosis but enhances survival by inducing *O*-GlcNAcylation and activating cytoprotective autophagy via AMPK. OSMI-1, which blocks *O*-GlcNAcylation and activates XBP1 to increase the autophagic flux, while activating IRE1α/JNK signaling. Therefore, combinatorial treatment with OSMI-1 and metformin significantly lowers the level of Bcl2 by combining the PERK/CHOP and IRE1α/JNK pathways and ultimately induces synergistic apoptosis.

## Discussion

Autophagy and apoptosis are closely linked to maintain cellular homeostasis even though they have different signaling pathways [21–23]. In general, autophagy and apoptosis inhibit each other; however, in some cases, autophagy promotes apoptosis [24–27]. It is known that the determinants of autophagy and apoptosis are highly dependent on the limits of cellular stress [28]. A recent report found that metformin inhibits the proliferation of colon cancer cells through autophagy and apoptosis, but the detailed mechanism of action of metformin during autophagy and its regulation through *O*-GlcNAcylation remain unclear [29, 30]. Therefore, in the present study, we investigated, both in vitro and in vivo, how inhibition of *O*-GlcNAcylation mutually contributes to autophagy and apoptosis in metformin-treated colon cancer cells.

Metformin is known to exert anti-aging effects by inhibiting mTOR in an AMPK-dependent and AMPK-independent manner [31, 32]. In the present study, we investigated the effects of metformin on apoptosis in colon cancer cells. Autophagy is one of the major mechanisms involved in cancer cell apoptosis and drug resistance in various cancer treatments [33–35]. We confirmed that AMPK inhibits mTOR, which negatively regulates autophagy, whereas inhibition of AMPK activity by Compound C partially blocked metformin-induced autophagy. Therefore, metformin treatment induced upregulation of autophagy through AMPK, resulting in elevated degradation of p62 and conversion of the autophagy marker LC3-I to LC3-II.

The activation of nutrient-sensitive pathways, such as OGT and AMPK, is closely related to cell proliferation and survival [36, 37]. *O*-GlcNAcylation is an important mechanism by which cells can respond to various stressors [38]. Hyper-*O*-GlcNAcylation also promotes cell proliferation and regulates cell survival in several cancers [39, 40]. The role of hyper-*O*-GlcNAcylation in metastasis and resistance to chemotherapy in cancers has been studied in-depth and regulation of *O*-GlcNAcylaion is a target for chemotherapy [41]. Chemotherapy-resistant breast cancer cell lines treated with the OSMI-1 exhibited anti-tumor activity through epigenetic activation of the tumor suppressor ERRFI1 [42]. Increased *O*-GlcNAc modification reduced ER stress-induced cell death, supporting the involvement of the HBP pathway in UPR activation [43, 44]. Several previous studies have established a link between the activation of *O*-GlcNAc modification and the induction of autophagy through ER stress. A recent study has demonstrated that IRE1α/XBP1 splicing is involved in the regulation of autophagy in HeLa cells [45]. The present study also showed that activation of the IRE1α/XBP1 axis mediates the increased expression of LC3B in response to OGT inhibition. OSMI-1 increased autophagic flux, but this effect was reversed in the presence of the IRE1α inhibitor 4µ8C. A recent study confirmed that autophagic flux activity is regulated by OSMI-1 using the LC3 turnover assay. These results suggest that inhibition of *O*-GlcNAcylation regulates ER stress (IRE1α)-induced autophagy in HCT116 cells.

On the other hand, elevated *O*-GlcNAcylation disrupts mitochondrial resistance, and ROS generation increases mitochondrial content by inhibiting lysosomal degradation [46]. In the present study, the levels of the autophagic markers LC3-II and SQSTM1/p62 were increased in HCT116 cells following *O*-GlcNAcylation inhibition, whereas Thiamet G treatment negatively regulated autophagy. We show here, for the first time, that metformin upregulates *O*-GlcNAcylation in HCT116 cells, which may be due to the prevention of cytotoxicity caused by ER stress. However, the exact molecular mechanisms by which metformin induces autophagy and activates *O*-GlcNAc, which is a negative mechanism for autophagy, require further study.

We also analyzed the roles of metformin and OSMI-1 in the ER stress pathway. The balance between survival and death due to ER stress and the UPR is determined by the duration and intensity of the stress. If persistent ER stress is not relieved, an increase in CHOP transcription factors may persist, leading to ER-mediated cell death [47, 48]. In the present study, metformin combined with OSMI-1 treatment increased the levels of ATF6, PERK, and IRE1α signaling pathways and also demonstrated a cytostatic effect in HCT116 cells. PERK activation and eIF2α phosphorylation are caused by metformin-mediated upregulation of CHOP in HCT116 cells. The major transcription factor CHOP is the most well-characterized proapoptotic pathway in the ER. In ER stress-induced apoptosis, CHOP promotes apoptosis by downregulating anti-apoptotic Bcl2 and upregulating the apoptotic mitochondrial factors BAX and BAK [49, 50]. In the present study, metformin-induced PERK/eIF2α/CHOP signaling did not significantly affect Bcl2 levels and thus did not induce cytochrome c release, which would lead to caspase-3 activation. The IRE1α pathway induces apoptosis through the activation of JNK but also alleviates ER stress through the induction of ER chaperones and ER stress-related degradation factors [51]. We also determined whether OSMI-1-induced autophagy and apoptosis were related to JNK signaling. Downregulation of OGT activation through OSMI-1 treatment promotes IRE1α activation in HCT116 cells, resulting in increased JNK phosphorylation. However, in the presence of OSMI-1, metformin-induced apoptosis in a concentration-dependent manner. In contrast, the SP600125 significantly inhibited OSMI-1-induced apoptosis in metformin-treated HCT116 cells. This suggests that apoptosis has a synergistic effect when both signaling pathways are combined. Taken together, these results indicate that metformin and OSMI-1 induce apoptosis by inhibition of Bcl2 and increasing cytochrome c release, thereby synergistically activating the PERK/CHOP and IRE1α/JNK signaling pathways, respectively.

The role of autophagy in cancer cells is multifaceted. On the one hand, it may be protective, but on the other hand, several anti-tumor studies have shown that cancer growth may be inhibited by autophagic cell death in various ways [52, 53]. Persistent cell damage remains and the aggravated autophagic flux can induce synergistic cell death instead of cytoprotective action [54]. In this study metformin triggered CHOP through ER stress in HCT116 cells, but did not ultimately lead to apoptosis by increasing *O*-GlcNAcylation and autophagy as a cytoprotective action. Inhibition of *O*-GlcNAcylation by OSMI-1 promoted IRE activation through ER stress, while inducing an increase in autophagy. On the other hand, combined treatment with metformin and OSMI-1 enhanced the autophagic pathway, but the combined ER stresses of both pathways eventually resulted in synergistic cell death. In cells treated with both metformin and OSMI-1, autophagic flux was greatly increased when metformin continuously induced autophagy through increasing AMPK activity and the homeostasis of *O*-GlcNAcylation was eliminated by OSMI-1. Further treatment with the autophagy inhibitor 3-MA, blocked the initial stages of autophagy, resulting in no activation of caspase-3. These results show that HCT116 cells induce autophagy at low levels or early stages of stress, whereas inducing apoptosis at a late stage or stress level above a threshold value. Thus, this suggests that the activation of apoptosis can be regulated by the level of autophagic flux and that autophagy and apoptosis are interconnected. A recent study showed that p62 accumulation triggered the activation of caspase-8 in HCT116 cells in the background of ABT263, an inhibitor of Bcl2 [55]. A previous study showed that TRAIL can induce the p62-dependent activation of caspase-8 and the accumulation of p62 can also promote caspase-8 activation [56]. Furthermore, activation of caspase-8 cleaves p62 protein, ultimately activating mTOR. This novel pathway of caspase-8-associated apoptosis was dependent on the autophagy-regulated proteins p62. In the present study, OSMI-1 also in-creased the expression level of p62, whereas the combination of metformin and OSMI-1 upregulated caspase-8 activity and cleaved p62 protein.

Cleavage of ATG5/7 inactivates the autophagic machinery in addition to producing pro-apoptotic protein fragments [57]. p62 is degraded by autolysosomes following the initiation of autophagy. Thus, cleaved p62 accumulation has been identified as a general marker of reduced autophagic flux [58]. A recent study reported that antioxidants and radiation cause caspase-mediated apoptosis owing to aberrant cleaved p62 accumulation, suggesting that cleaved p62 accumulation is associated with autophagic flux–associated cytotoxicity through inhibition of *O*-GlcNAcylation [59]. Therefore, these results suggest that the activation of apoptosis signaling induces blockade or accumulation of the autophagic flux, resulting in a synergistic increase in apoptosis. Taken together, the degree of apoptosis of HCT116 cells upon treatment with a combination of metformin and OSMI-1 was modulated according to the level of autophagic flux, whereas activation of apoptosis also regulated the level of autophagic flux. Autophagy and apoptosis mostly appear to inhibit reciprocally, but the exact molecular mechanism underlying this requires further study.

## Conclusions

In conclusion, we showed that metformin induces autophagy in response to ER stress in HCT116 cells. Metformin increases P-AMPK levels and activates autophagic flux and also induces *O*-GlcNAcylation, which exerts a cytoprotective function. Treatment with OSMI-1, which inhibits *O*-GlcNAcylation, activates autophagic flux along with the ER stress response. Combination treatment with metformin and OSMI-1 synergistically induced apoptosis in HCT116 cells. The combination of metformin and OSMI-1 that induce autophagy may have important clinical benefits. Previous studies have demonstrated that the inhibition of OGT activity also increases sensitivity to several other chemotherapies. Therefore, combination therapy with metformin and OSMI-1 is expected to be successfully applied for the treatment of colon cancer cells, as well as other cancer types.

## Supplementary Information

**Additional file 1: Figure S1.** Confirmation of autophagy induction through AMPK in HCT116 cells. **Figures S2–S3.** Effect of cell death and *O*-GlcNAcylation on metformin-induced SW620 cells. **Figure S4.** Effect of *O*-GlcNAcylation on OSMI-1-induced HCT116 cells. **Figure S5.** IRE1α pathway by metformin. **Figure S6.** JNK pathway by metformin. **Figure S7.** Western blot analysis of ER stress markers. **Figure S8.** Effects of combined treatment with metformin and OSMI-1 in HCC cell lines HepG2 and Huh7 cells. **Figure S9.** Synergistic effects of combined treatment with metformin and OSMI-1 in HCT116 cells. **Figure S10.** Inhibition of growth and apoptosis of xenograft tumors by combination treatment of metformin and OSMI-1 in HCT116 p53^+/+^. **Figure S11.** Inhibition of growth and apoptosis of xenograft tumors by combination treatment of metformin and OSMI-1 in HCT116 p53^−/−^.

## Data Availability

The data presented in this study are available on request from the corresponding author. The data are not publicly available due to privacy.

## Abbreviations

4PBA: 4-Phenyl butyric acid
AMPK: Adenosine monophosphate-activated protein kinase
ATF6: Transcription factor 6
CHOP: C/EBP homologous protein
DMEM: Dulbecco’s modified eagle medium
DMSO: Dimethyl sulfoxide
ER: Endoplasmic reticulum
FACS: Fluorescence-activated cell sorting
FBS: Fetal bovine serum
GFAT: Glutamine:fructose-6-phosphate amidotransferase
HRP: Horseradish peroxidase
IF: Immunofluorescence
IRE1α: Inositol-requiring enzyme 1 α
JNK: C-Jun N-terminal kinase
LC3: The microtubule-associated proteins 1A/1B light chain 3 B
mTOR: Mammalian target of rapamycin
OGA: *O*-GlcNAcase
OGT: *O*-GlcNAc transferase
PE: Phosphatidylethanolamine
PERK: Protein kinase RNA-like endoplasmic reticulum kinase
RT-PCR: Reverse transcription-polymerase chain reaction
SDS: Sodium dodecyl sulfate
SDS-PAGE: SDS-polyacrylamide
sXBP1: Splicing level of XBP1
UPR: Unfolded protein response
