# Supplementary material for: Synergistic apoptosis by combination of metformin and an O-GlcNAcylation inhibitor in colon cancer cells
Source: Cancer Cell Int. 2023 Jun 2;23:108. doi: 10.1186/s12935-023-02954-2 (PMC10239094; doi:10.1186/s12935-023-02954-2)
Supplement: Supplementary file 1 — Additional file 1: Figure S1. Confirmation of autophagy induction through AMPK in HCT116 cells. Figures S2–S3. Effect of cell death and O-GlcNAcylation on metformin-induced SW620 cells. Figure S4. Effect of O-GlcNAcylation on OSMI-1-induced HCT116 cells. Figure S5. IRE1α pathway by metformin. Figure S6. JNK pathway by metformin. Figure S7. Western blot analysis of ER stress markers. Figure S8. Effects of combined treatment with metformin and OSMI-1 in HCC cell lines HepG2 and Huh7 cells. Figure S9. Synergistic effects of combined treatment with metformin and OSMI-1 in HCT116 cells. Figure S10. Inhibition of growth and apoptosis of xenograft tumors by combination treatment of metformin and OSMI-1 in HCT116 p53+/+. Figure S11. Inhibition of growth and apoptosis of xenograft tumors by combination treatment of metformin and OSMI-1 in HCT116 p53−/−. [file 12935_2023_2954_MOESM1_ESM.docx]

**Additional file 1**


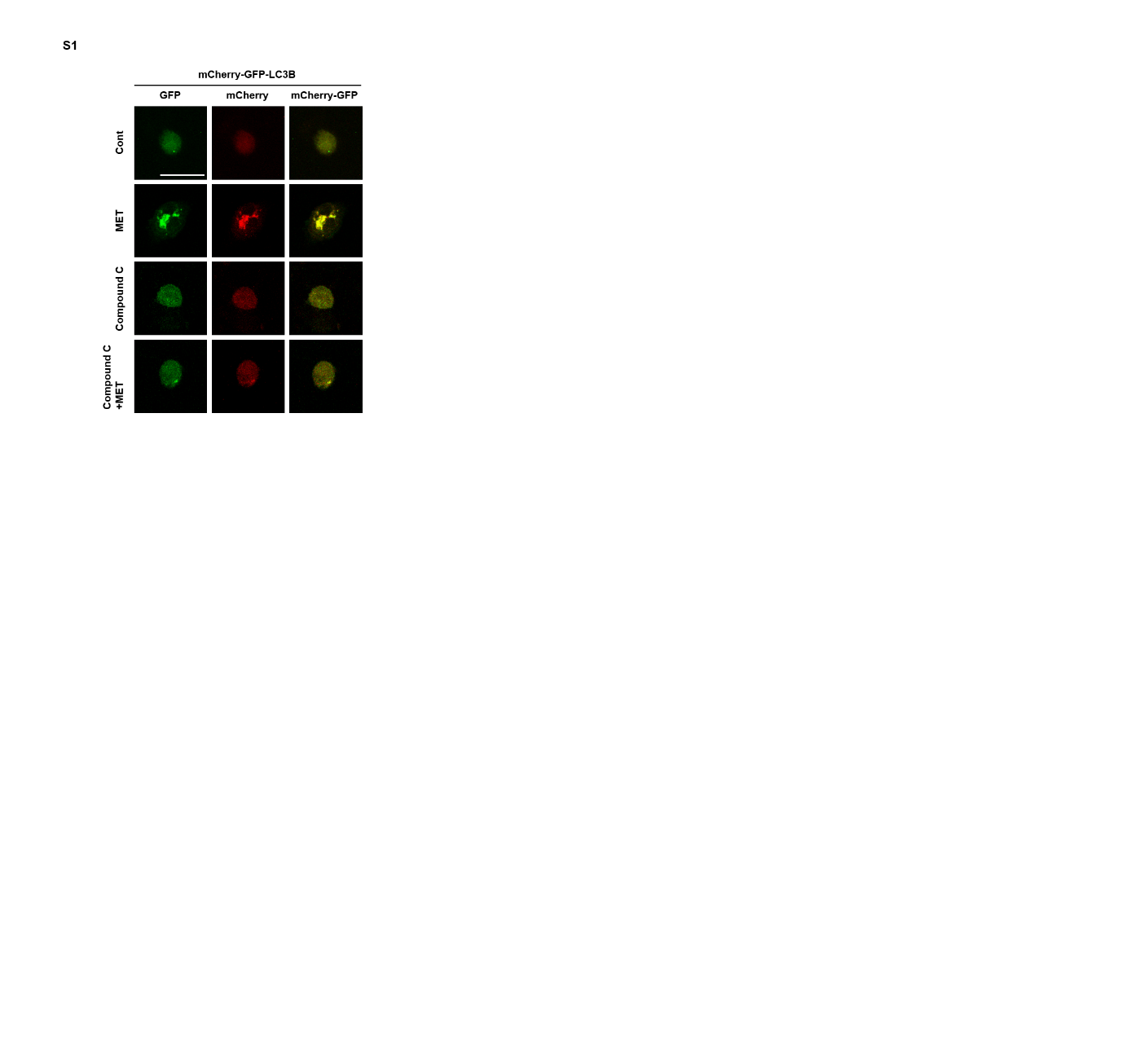


**Fig. S1 Confirmation of autophagy induction through AMPK in HCT116 cells** HCT116 cells were transfected with the mCherry-EGFP-LC3 plasmid for 24 h and treated with metformin (25 mM) for 48 h. The Compound C treated group was transfected with the plasmid and then pretreated with Compound C (20 μM) for 1 h, followed by treatment with metformin (25 mM) for 48 h. Immunofluorescence staining of LC3 was visualized using a confocal laser scanning microscope (original magnification ×1000, scale bar = 10 μm).


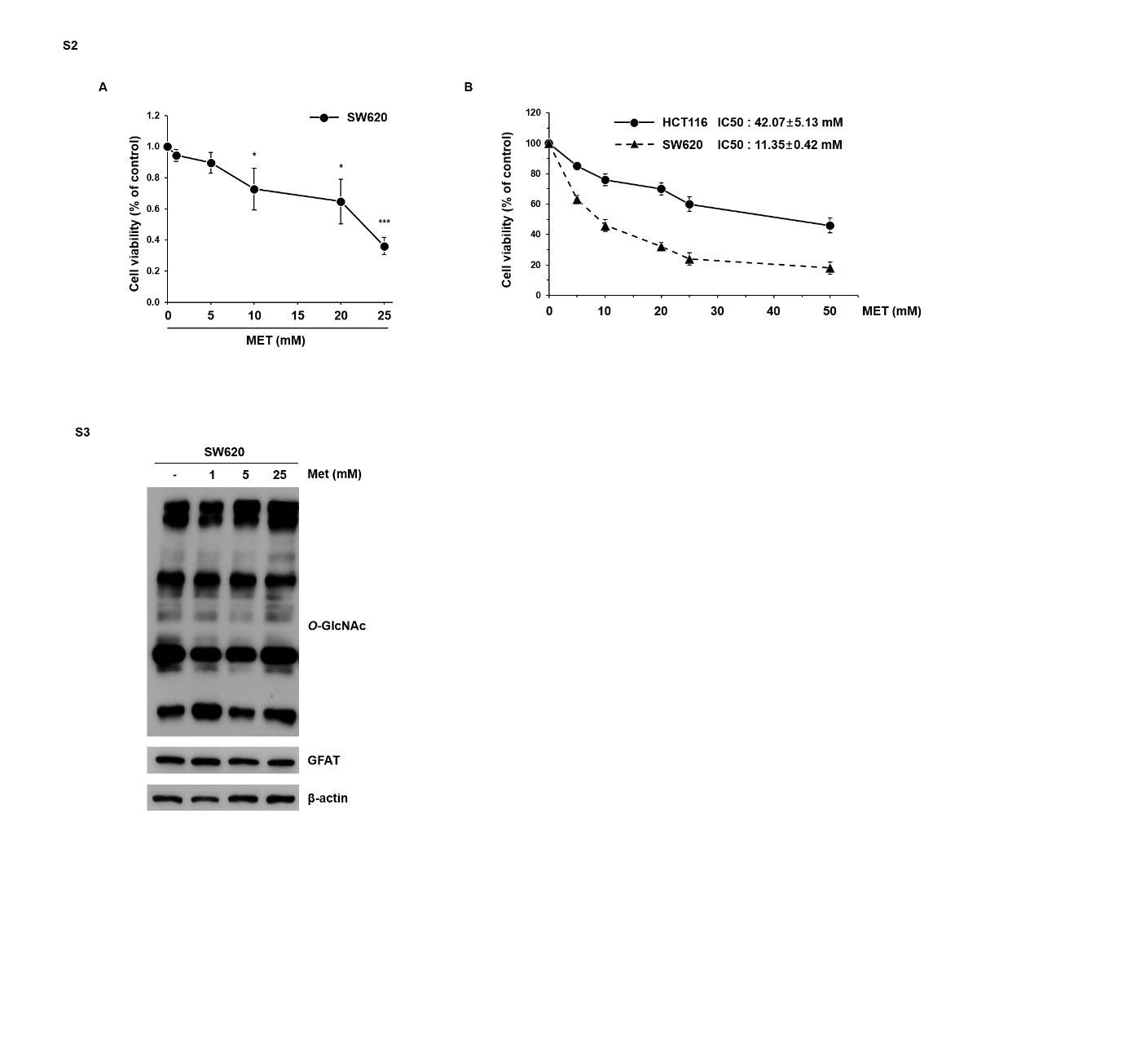

**Fig. S2 and S3 Effect of cell death and *O*-GlcNAcylation on metformin-induced SW620 cells** (S2A) SW620 cells were incubated with metformin (1, 5, 10, 20, and 25 mM) for 48 h. Cell viability was analyzed using the MTT assay. (S2B) HCT116 and SW620 cells were incubated with metformin (5, 10, 20, 25, and 50 mM) for 72 h. Cell viability was determined by MTT assay and data shown are the measured IC50 value. (S3) SW620 cells were treated with different concentrations of metformin (1, 5, and 25 mM) for 48 h. The levels of *O*-GlcNAc and GFAT were examined by western blot analysis. β-actin was used as a loading control in western blot analyses.


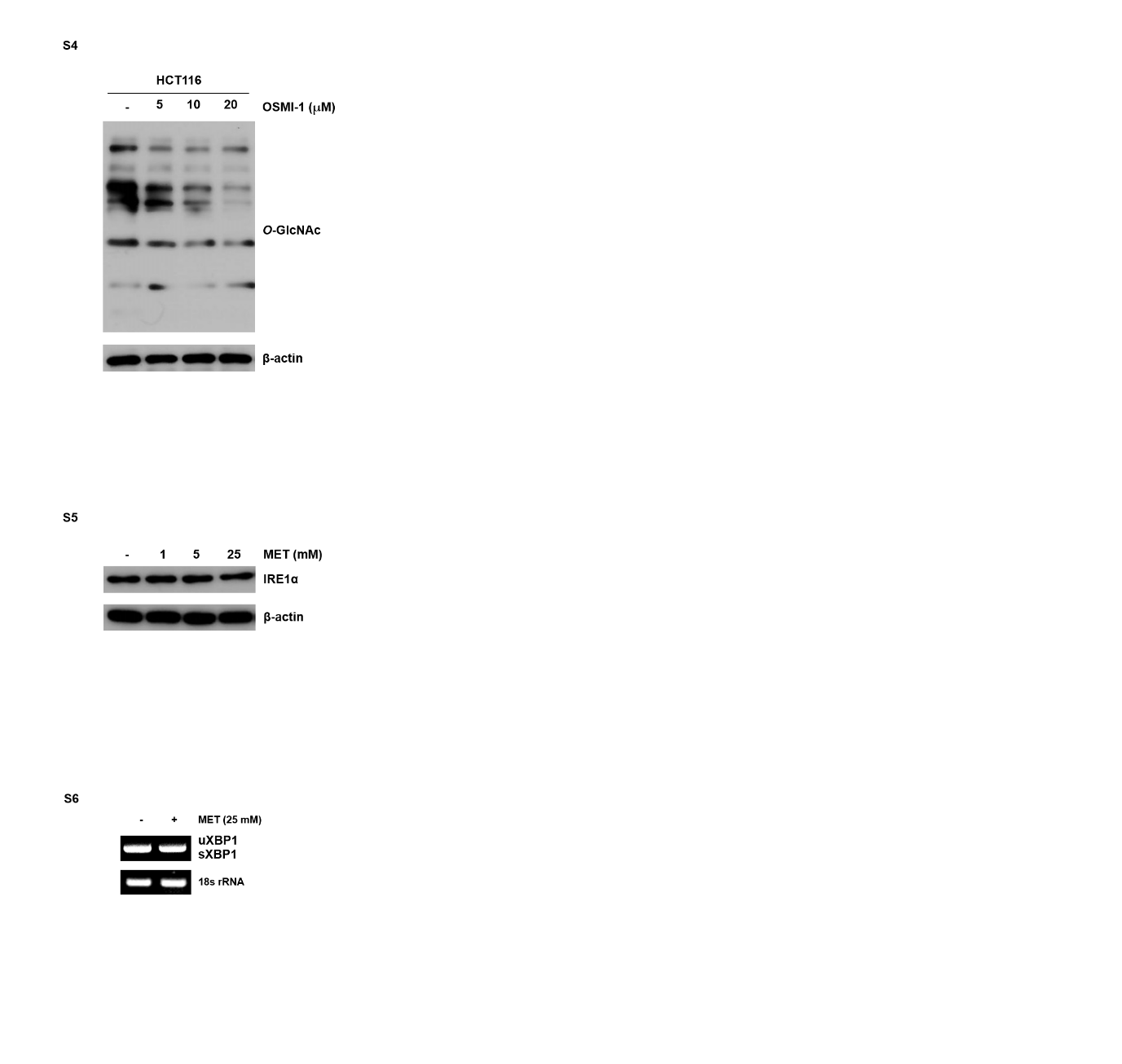


**Fig. S4 Effect of *O*-GlcNAcylation on OSMI-1-induced HCT116 cells** HCT116 cells were treated with different concentrations of OSMI-1 (5, 10, and 20 μM) for 48 h. The level of *O*-GlcNAc was examined by western blot analysis. β-actin was used as a loading control in western blot analyses.


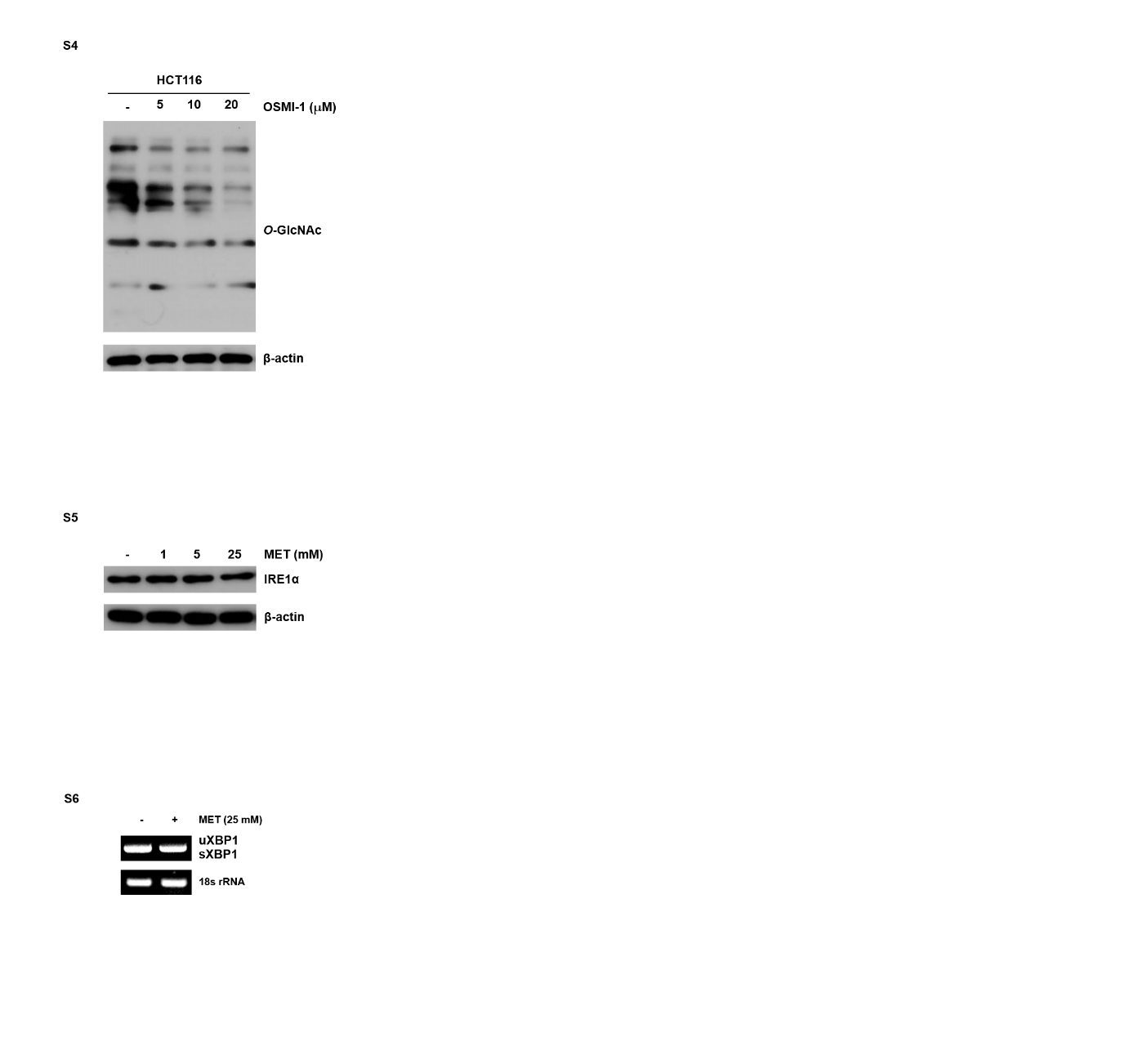


**Fig. S5 IRE1α pathway by metformin** HCT116 cells were treated with metformin (25 mM) for 48 h. The levels of IRE1α were examined by western blot. β-actin was used as a loading control in western blot analyses.


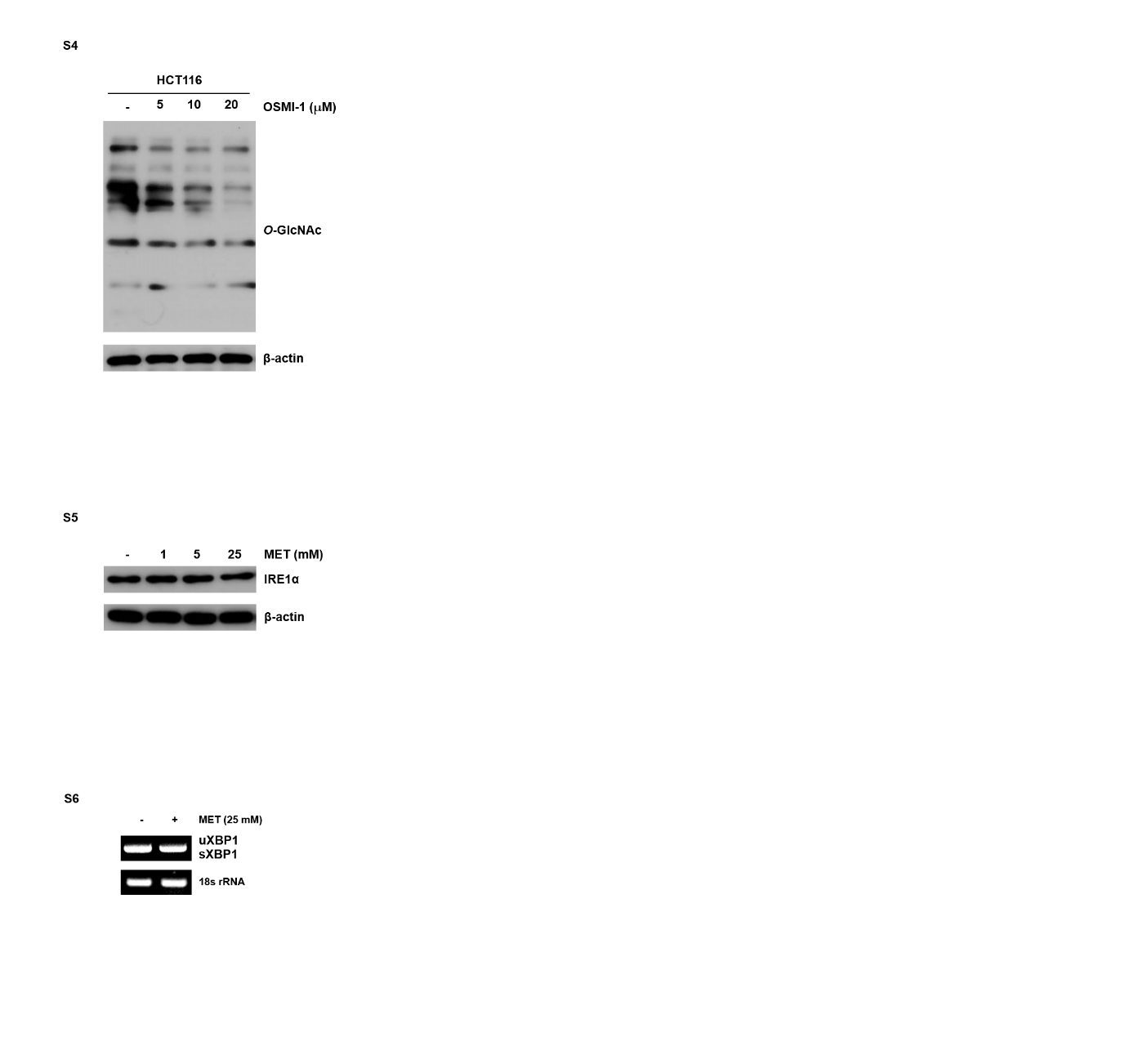


**Fig. S6 JNK pathway by metformin** HCT116 cells were treated with metformin (25 mM) for 48 h. The levels of XBP1 were analyzed by RT-PCR. 18s rRNA was used as a loading control in RT-PCR.


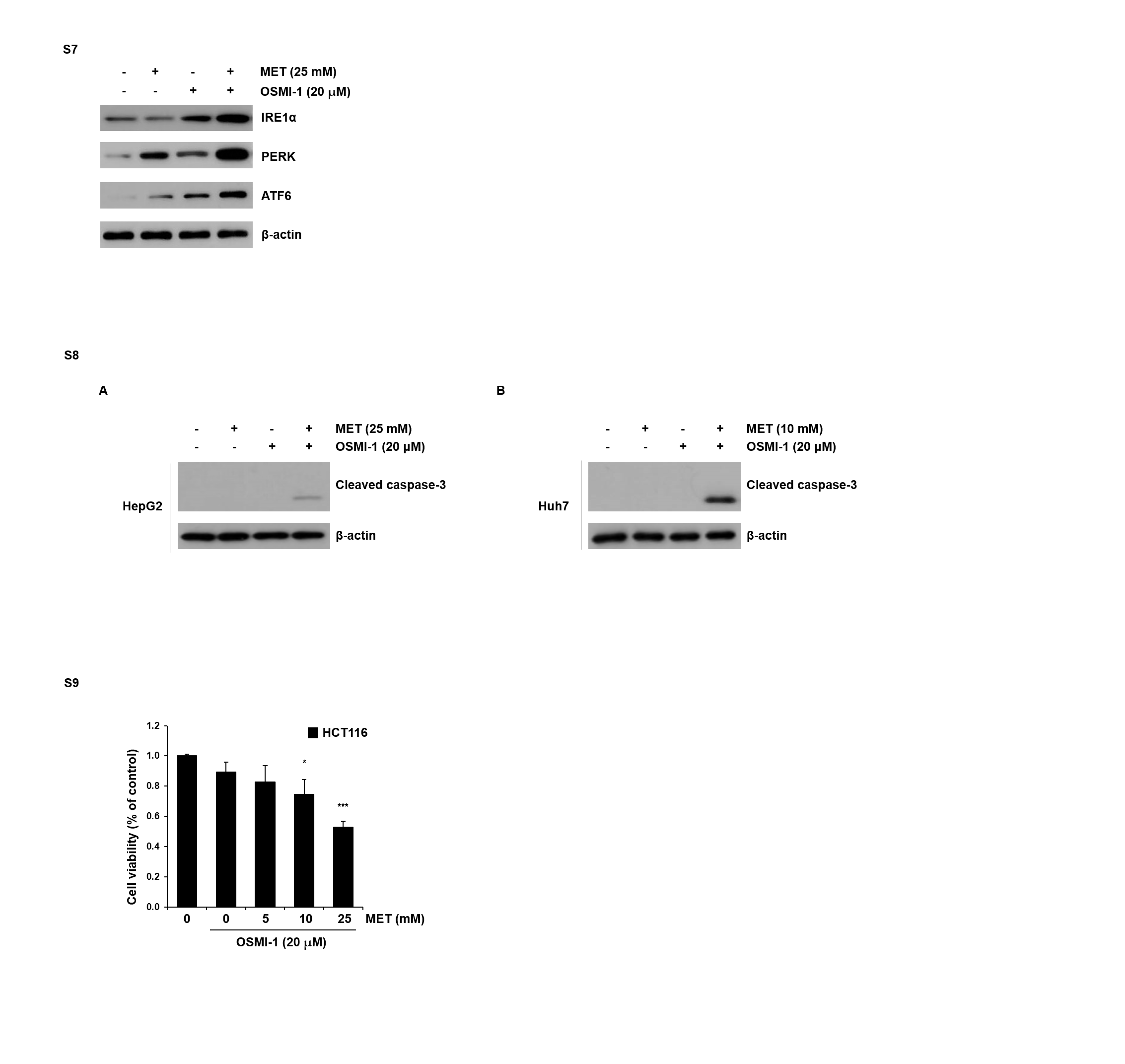


**Fig. S7 Western blot analysis of ER stress markers** HCT116 cells were treated with metformin (25 mM) and OSMI-1 (20 μM) alone or in combination for 48 h. The levels of IRE1α, PERK, and ATF6 were examined by western blot. β-actin was used as a loading control in western blot analyses.


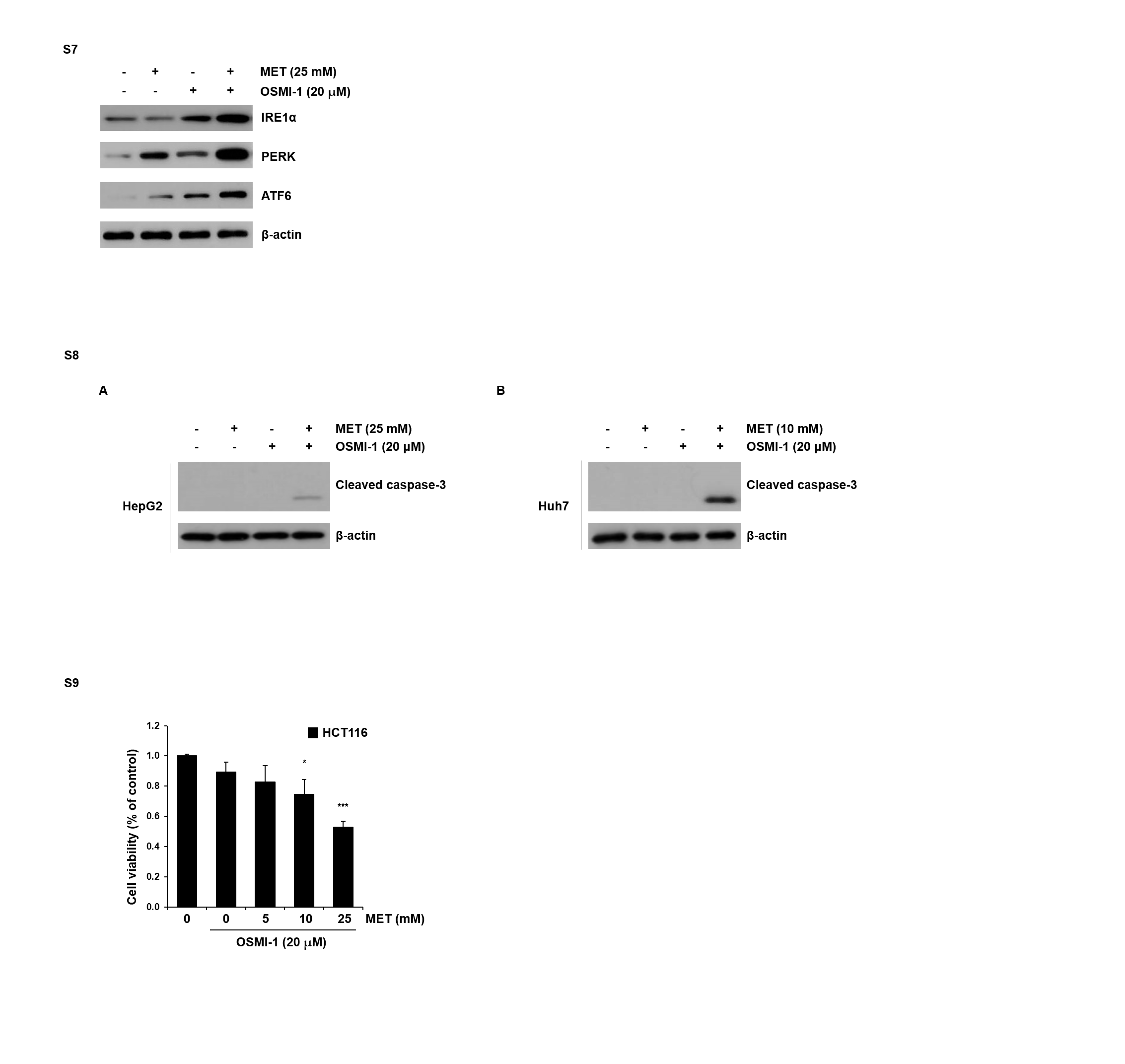


**Fig. S8 Effects of combined treatment with metformin and OSMI-1 in HCC cell lines HepG2 and Huh7 cells** (A) HepG2 cells were treated with OSMI-1 (20 μM) and metformin (25 mM) alone or a combination thereof for 24 h. The level of cleaved caspase-3 was determined by western blot analysis. (B) Huh7 cells were treated with OSMI-1 (20 μM) and metformin (10 mM) alone or a combination thereof for 24 h. The level of cleaved caspase-3 was determined by western blot analysis. β-actin was used as a loading control in all western blot analyses.


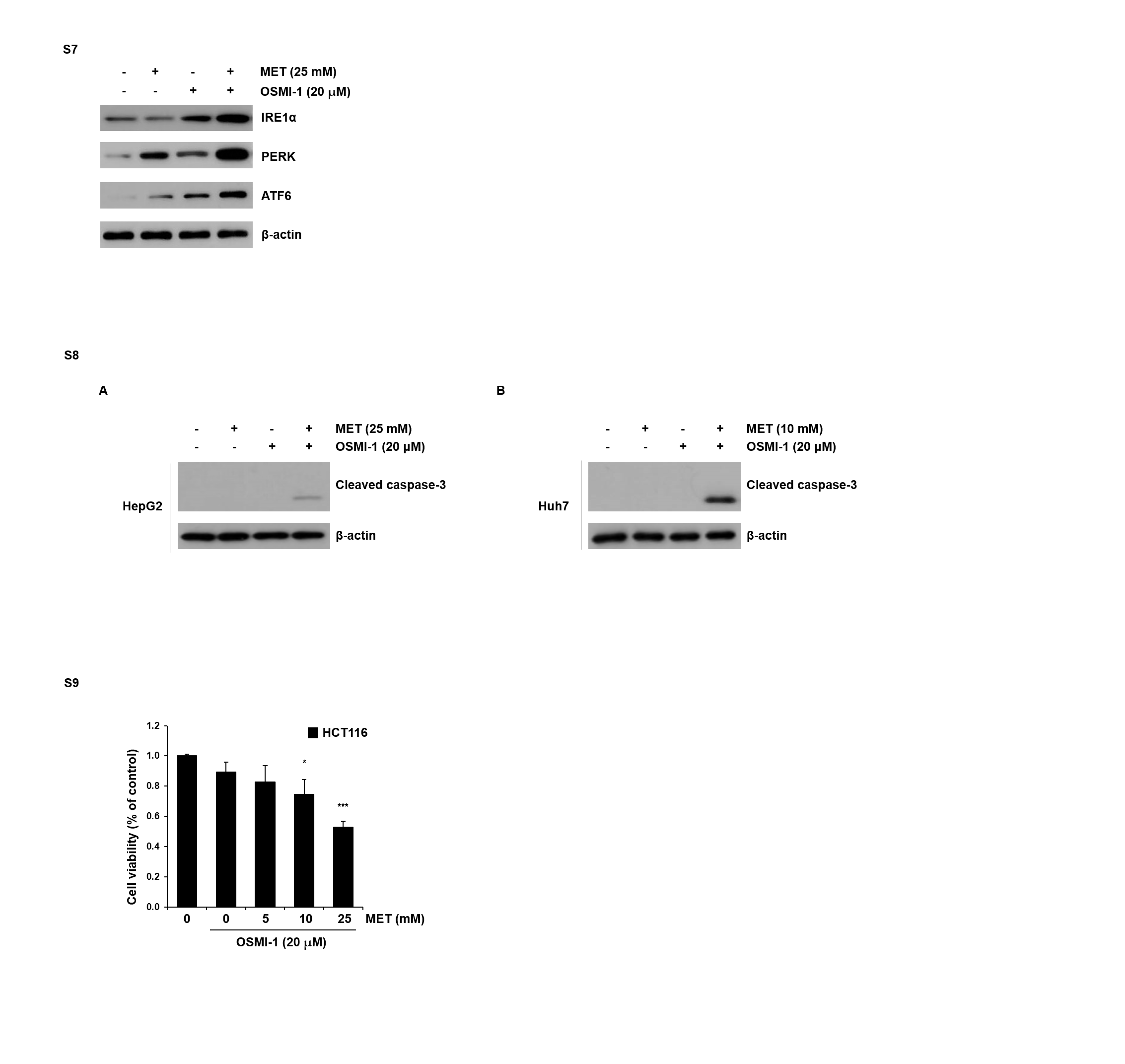


**Fig. S9 Synergistic effects of combined treatment with metformin and OSMI-1 in HCT116 cells** HCT116 cells were treated with OSMI-1 (20 μM) and metformin (5, 10, and 25 mM) for 48 h, and the cell viability was determined by MTT assay. Significance was determined by control and combination group Student’s t-test. * *p* < 0.05, *** *p* < 0.001.


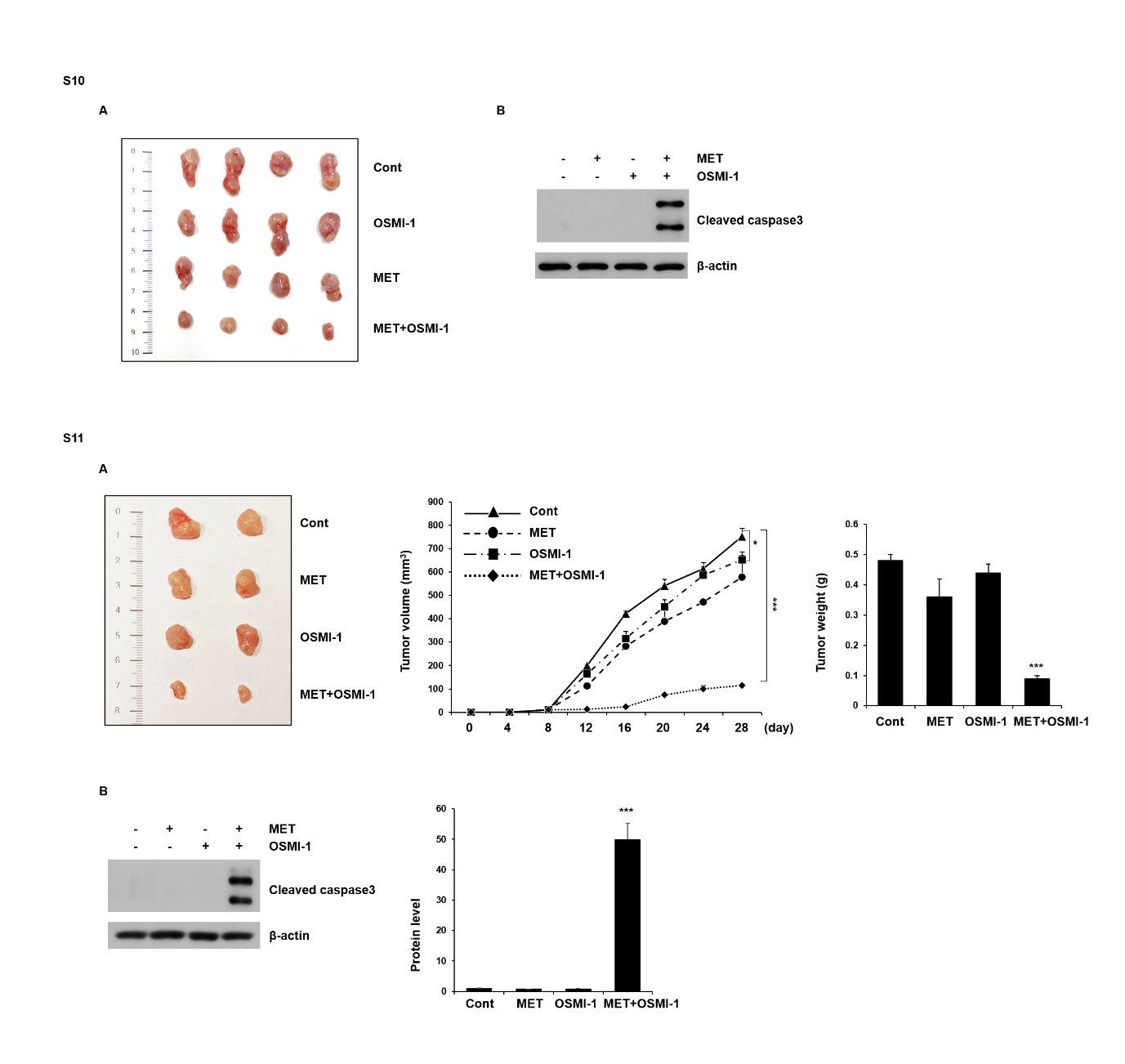

**Fig. S10 Inhibition of growth and apoptosis of xenograft tumors by combination treatment of metformin and OSMI-1 in HCT116 p53^+/+^** (A) BALB/c nude female mice were inoculated subcutaneously with HCT116 p53^+/+^ cell administration vehicle (DMSO), metformin, OSMI-1 or a combination and tumor growth was monitored for 28 days. Representative images of subcutaneous xenograft tumors in nude mice treated with metformin, OSMI-1, or a combination. (B) Expression levels of cleaved caspase-3 involved in apoptosis were also determined by western blot, and β-actin was used as the loading control.


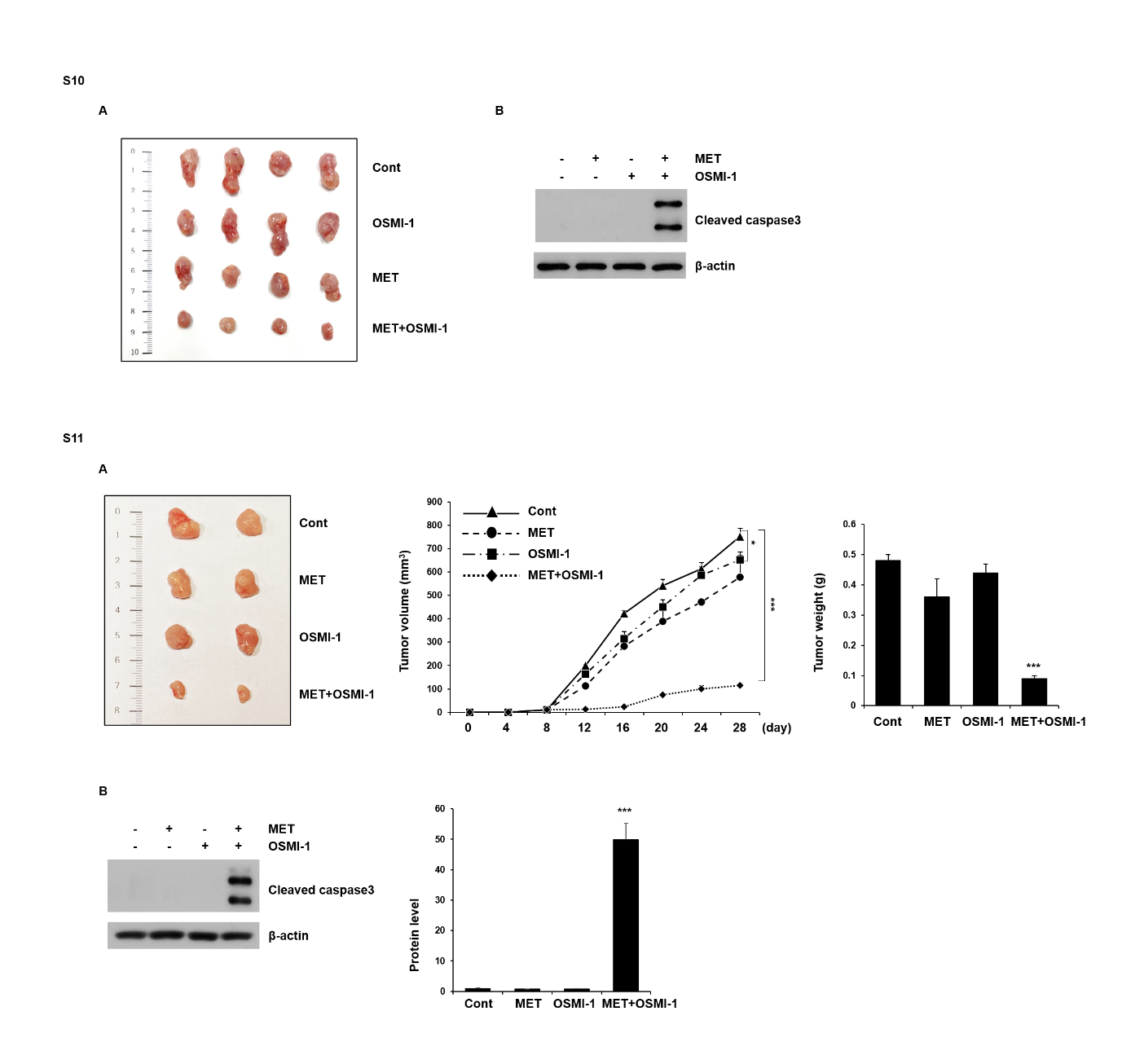

**Fig. S11 Inhibition of growth and apoptosis of xenograft tumors by combination treatment of metformin and OSMI-1 in HCT116 p53^-/-^** (A) BALB/c nude female mice were inoculated subcutaneously with HCT116 p53^-/-^ cell administration vehicle (DMSO), metformin, OSMI-1 or a combination and tumor growth was monitored for 28 days. Representative images of subcutaneous xenograft tumors in nude mice treated with metformin, OSMI-1, or a combination (right). Volumes of xenograft tumors in metformin, OSMI-1, and combination treatment groups at the indicated time points were measured and growth profiles were expressed as mean ± SEM (middle). Histograms are the results of tumor weight measurements at endpoints and are presented as mean ± SEM (right). (B) Expression levels of cleaved caspase-3 involved in apoptosis were also determined by western blot, and β-actin was used as the loading control. (A, B) Significance was determined by control and combination group Student’s t-test. * *p* < 0.05, *** *p* < 0.001.
